# Are the estimated needs for mental health care among adolescents from different socioeconomic backgrounds met equally in Sweden? A longitudinal survey-registry linkage study

**DOI:** 10.1007/s00787-023-02341-2

**Published:** 2023-12-29

**Authors:** Joseph Jr. Muwonge, Christina Dalman, Bo Burström, Antonio Ponce de Leon, Maria Rosaria Galanti, Beata Jablonska, Anna-Clara Hollander

**Affiliations:** 1 Centre for Epidemiology and Community Medicine, Stockholm, Sweden; 2 Department of Global Public Health, Karolinska Institute, Stockholm, Sweden

**Keywords:** Need for care, Inequity, Children, Youth, Adolescent mental health

## Abstract

Evidence of inequality in the utilisation of mental health care (MHC) by adolescents in Nordic countries is mixed. This study aims to investigate if there are socioeconomic differences in the utilisation of MHC, while accounting for adolescents’ mental health status. We analysed a cohort of 3517 adolescents, followed from 7 to 9th grade (ages 13–16), to examine the association between parental socioeconomic position (SEP: education and disposable income), adolescents’ estimated needs, and the utilisation of MHC (defined as visits to secondary psychiatric care or receipt of psychotropic medication). Logistic and negative binomial regression models, with mental health status as moderator, were used to predict utilisation during each grade. Lower SEP predicted higher odds of utilising MHC in adolescents with no/mild symptoms (e.g., odds ratio, OR = 1.33, 95% CI 1.04–1.72, lower vs highest education), but not in those with moderate-to-severe symptoms (estimates close to one and non-significant). This pattern was largely explained by treatment of attention deficit hyperactivity disorder/autism spectrum disorders (ADHD/ASD) in boys. For girls with severe symptoms, lower SEP predicted reduced odds of utilising MHC for other mental disorders (OR = 0.48, 95% CI 0.25–0.92, lower education), and fewer outpatient visits when in contact with such care, although non-significant (incidence rate ratio, IRR = 0.51, 95% CI 0.25–1.05, lowest vs highest income). Our findings suggest a more equitable use of MHC for treating ADHD/ASD, but not other mental disorders such as depression and anxiety, particularly among girls.

**Supplementary Information:**

The online version contains supplementary material available at 10.1007/s00787-023-02341-2.

## Introduction

Adolescence is a critical period in life when mental disorders often debut [1]. Between 14 and 31% of adolescents globally are estimated to suffer from at least one mental disorder [2, 3]. Since there is a social gradient in mental disorders, where adolescents from families of lower socioeconomic position (SEP) are more likely to suffer from mental disorders than their counterparts from families of higher SEP [4, 5]. It would be expected that this translates to higher utilisation of mental health care (MHC) in adolescents of lower SEP [6]. However, the processes involved in seeking and using care also vary by SEP. In addition to differences in the recognition of symptoms and perception of need [7], factors, such as cost, proximity to care, and ability to navigate the health care system, could influence the translation of perceived need for care to actual demand for care [7–10]. Furthermore, when in contact with care, differences in user-provider interactions, such as communication of symptoms, and differences in compliance to care, might explain the observed socioeconomic inequality in the utilisation of MHC [7, 10]. Overall, socioeconomic differences in care seeking processes could lead to disparity in terms of adolescents contacting care and the amount of care used. Adolescents’ SEP can be conceptualised among other ways as parents’ educational attainment and disposable household income. These factors are markers of parents’ health literacy, who are often involved in their children’s care seeking [11], power to influence health outcomes, access to material resources, and social structures, including safer neighbourhoods and social capital, which are important for wellbeing and accessing health care when needed [12, 13].

Evidence of inequality in the utilisation of MHC by adolescents in Nordic countries, where the healthcare system is universal, is mixed. Some studies indicate that adolescents from families of lower SEP use less MHC than adolescents from families of higher SEP [14], whereas other studies show the opposite [15–17], or no differences in the use of MHC [18]. One significant limitation with previous studies is that few have investigated both the need for and the utilisation of MHC. Need for care should ideally influence use of MHC and it is therefore important to be considered when studying differences in the utilisation of MHC.

Therefore, this study aims to investigate if there are socioeconomic differences in the utilisation of MHC at least once, and in the number of outpatient visits in adolescents, when considering their mental health status. Our hypothesis is that SEP might predict the utilisation of MHC among adolescents differently depending upon the severity of symptoms.

## Methods

### Design

This is a secondary analysis of data from a population-based longitudinal study conducted between 2013 and 2018 (Kupol study) [19].

### Setting

Sweden has a decentralized healthcare system where financing and provision of health care is the responsibility of the 21 county councils (nowadays called regions) [20]. MHC is mostly provided by publicly funded facilities. Primary care provides early interventions (children also receive help through school health services) and acts as a gatekeeper to secondary care. First-line MHC services, introduced in 2014, are meant to handle milder cases in primary care or secondary care facilities (depending upon the region) to prevent unnecessary contact with specialized care [21]. Children with more severe conditions are referred to secondary care, typically to Child and Adolescent Psychiatry (CAP); however, there is an open-door policy in CAP where children or their guardians can contact CAP without referrals. CAP has both outpatient and inpatient services; however, 98% of all individuals in contact with CAP use outpatient services. About 30% of all visits to CAP are for ADHD treatment (2022) [22]. Diagnosis of Autism Spectrum disorders (ASD) occurs in secondary care (primarily at CAP), after which adolescents receive help from both secondary care and rehabilitation services [23].

Timely access to services is promised under the so-called “Healthcare guarantee”, which is a national policy under chapter 2 of the Patient Act [24, 25]. This policy guarantees same-day telephone contacts and an examination by a licensed provider within three days of first contact. Children are guaranteed a first visit to CAP within 30 days of contact/referral. Finally, health care is free for children under 18 except at emergency departments, where a user-fee of 120 Swedish crowns (SEK; approximately 10 euros) is charged.

### Population

**Fig. 1:**
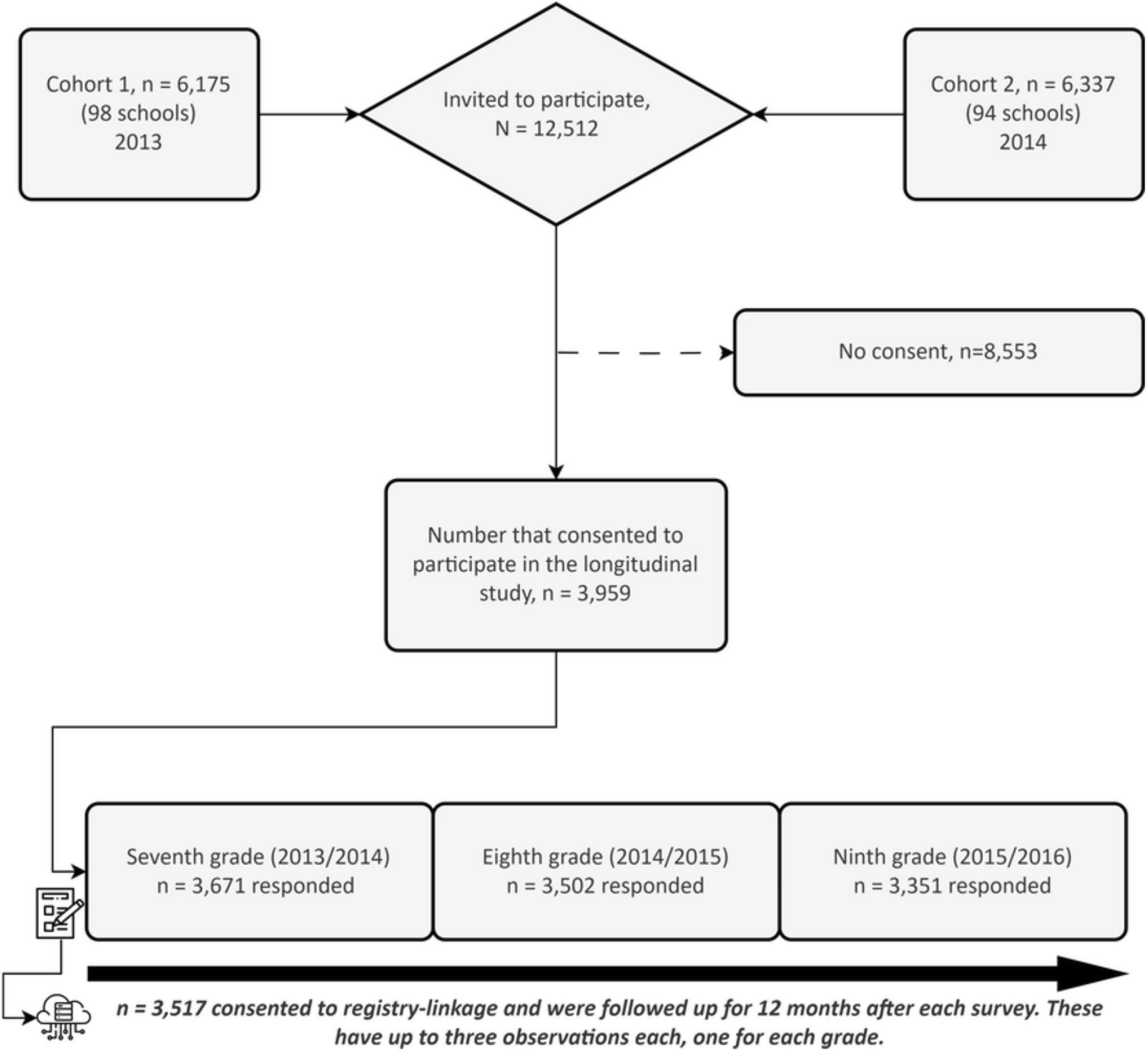
Information about the recruitment of the cohort

A total of 12,512 adolescents of ages 13–14 years who were in 7th grade in 2013 (cohort 1) or in 2014 (cohort 2) were invited to participate in the longitudinal study. These students were from 101 schools located in eight regions of southern and central Sweden, namely, Gävleborg, Jönköping, Stockholm, Södermanland, Uppsala, Värmland, Västmanland, and Örebro. Parents of 3959 adolescents consented to participate, of which parents to 3517 adolescents agreed to registry-linkage and constitute the analysed sample. See Fig. 1.

### Data materials

The Kupol project (Swedish acronym for “*Kunskap om ungas psykiska hälsa och lärande”*) collected information primarily through surveys, with subsequent record-linkage of survey data with healthcare registries (see under Variables) using personal identification numbers. Surveys were collected yearly (at school and at home), from 7th grade until 1st year of secondary school, that is, four repeated surveys.

The survey-registry linkage enabled us to access information on adolescents’ mental health status, an indicator of need for care from the surveys, and information on adolescents’ healthcare records from administrative registries. For this study, we have used the data in 7th, 8th, and 9th grade (when adolescents were 13–16 years of age) where we had registry data.

Detailed information about the Kupol project aims, and recruitment of schools and students has been published elsewhere [19, 26].

### Variables

#### Outcome variable: Utilisation of MHC

Information about the adolescents’ utilisation of MHC was collected from the National Patient Registry (NPR; from the National Board of Health and Welfare), the registries from the Child and Adolescent Psychiatry clinics (CAP; *Barn och Ungdomspsykiatri* in Swedish), and the Prescribed Drugs Registry containing data on prescriptions given either at primary or secondary care (PDR; from the National Board of Health and Welfare).

We defined two outcome variables:

- Utilised MHC at least once: We identified adolescents who had at least one visit to secondary psychiatric care (including outpatient and inpatient care, as well as all utilisation in regional CAP clinics), or who received prescribed psychotropic drugs during 12 months after each survey, in 7th–9th grade. Diagnoses were defined according to the International Classification of Diseases, tenth revision (ICD-10), using the following codes: F0-F99, G47, X60-X84, Z91.5, R45, Z72.820, Z73.3, Z73.4, Z73.9, Z72.810, and Z032. Psychotropic drugs were defined according to the Anatomical Therapeutic Chemical code (ATC), using the following codes: N05A, N05B, N05C, N06A, and N06B.
- Number of visits: number of outpatient visits to secondary psychiatric care among adolescents in contact with any MHC service during follow-up at each grade. We counted one visit per date even when an individual might have had more than one visit on the same date.

Since healthcare seeking patterns and use might differ between adolescents with neuropsychiatric conditions and those with other mental disorders [27], we have further categorised the outcome variables into the following subgroups:

- Attention-deficit/hyperactivity disorder or autism spectrum disorders (ADHD/ASD): This sub-group includes visits to in- and outpatient secondary psychiatric care with a recorded diagnosis of ADHD or ASD, or the receipt of ADHD medicine (ATC N06B). See Table s1 in the supplementary material for additional details.
- Other mental disorders: This sub-group includes adolescents who used MHC for mental disorders other than ADHD/ASD, such as depression and anxiety.

#### Exposure variables: socioeconomic position (SEP)

Information about the parents’ education level and equivalized disposable household income was collected from the longitudinal integration database for Health Insurance and Labor Market Studies (LISA by Swedish acronym) at Statistics Sweden (SCB *by Swedish acronym*).

- Parents’ education: Parents’ highest attained educational level was categorised into both parents without tertiary education (≤ 12 years of school) and at least one parent with tertiary education (≥ 13 years of school).
- Household income: Equivalised/weighted disposable household income, estimated by Statistics Sweden (SCB) for each household every year, was categorised into tertiles, i.e., lowest, middle, and highest income.

For both parents’ education and household income, we used figures for the year preceding the calendar year when MHC utilisation was measured; for instance, household income in 2013 was used to predict utilisation in 2014.

#### Moderating variable: adolescents’ mental health status

Information about adolescents’ mental health status, measured by the self-rated and parent-rated Strengths and Difficulties Questionnaire (SDQ), was collected by surveys at each grade. The SDQ instrument is validated to identify Swedish children and adolescents (1–19 years) with mental health problems [28–30]. Children and adolescents identified to have mental health problems using the SDQ are more likely to seek professional help [31], and/or to receive a psychiatric diagnosis [32]. The SDQ instrument contains five sections on prosocial behaviour, hyperactivity-inattention, emotional symptoms, conduct problems, and peer problems. The last four sections are summed to create the Total difficulties score (with a symptom score range of 0–40). We applied previously used cut-off points [19] to categorise adolescents into three groups based on their Total difficulties scores. Using the self-rated SDQ instrument, we categorised adolescents who scored 0–15 as having “no/mild symptoms”, those who scored 16–19 as having “moderate symptoms”, and those who scored 20–40 as having “severe symptoms”.

#### Covariates

Adolescent’s sex was reported at baseline as girl or boy. Sex was included as a covariate, since there are sex-based differences in both reported mental health problems and the use of MHC. To account for secular variations in the use of MHC, we used the calendar year when MHC utilisation was measured: 2014, 2015, & 2016 for cohort 1 and 2015, 2016, & 2017 for cohort 2.

In addition, region of residence at each grade included: Gävleborg, Jönköping, Stockholm, Södermanland, Uppsala, Värmland, Västmanland, and Örebro; we adjusted for regions, because we anticipated regional variations in the use of MHC.

Parent’s country of birth was self-reported at baseline and categorised as having at least one parent born in Sweden or both parents (or the “single” parent) born outside Sweden. Parental mental illness, collected from the NPR, was dichotomised as “yes” if either parent had ever received treatment for a mental disorder in secondary care (measured at each grade), and “no” otherwise. Both parent’s country of birth and parental mental illness were considered confounding factors in the relationship between SEP and MHC utilisation in adolescents.

### Statistical analysis

A logistic regression analysis was performed to estimate the association between SEP and the utilisation of MHC at least once during 12 months after each survey (3 surveys). The repeated subject statement was used in procedure GENMOD [Generalized Estimating Equations (GEEs) models], with an exchangeable correlation structure, to account for nesting of observations within participants (repeated surveys) [33]. Models allowing for interaction effects were reparametrized to estimate odds ratios of the association between SEP and the use of MHC in each category of adolescents’ mental health status (“no/mild”, “moderate”, and “severe” symptoms; see supplementary Table s2 for the syntax). These models were adjusted for calendar year, region of residence, parent’s country of birth, and parental mental illness (and adjusted for sex in unstratified models). Odds ratios (OR) with their corresponding 95% confidence intervals are reported.

Among adolescents in contact with any MHC, a negative binomial regression was performed, to estimate the association between SEP and the number of outpatient visits during 12 months after each survey. Models allowing for interaction terms between SEP and adolescents' mental health status were performed while accounting for repeated observations (like the procedure above). These models were also adjusted for the covariates listed above. Incidence rate ratios (IRR) with their corresponding 95% confidence intervals are reported.

### Handling missing values

Over the three grades, the proportion of missing data was low for most of the key variables (< 3%) except for missing data on mental health status (10%). Since parental education level is stable in this group (mean age 45), we replaced the missing on education with information collected in another grade if available. A similar approach was not possible for missing on household income and self-reported mental health status, since these measurements could be unstable over the three grades. Therefore, the main analysis was based on complete cases.

### Sensitivity analysis

Due to the potential clustering of students within schools, we ran a three-level hierarchical model for clustering of observations within students and clustering of students within schools using procedure GLIMMIX (hierarchical generalized linear models) in SAS. We calculated the intraclass correlation coefficients (ICC) based on estimates produced from empty models predicting the utilisation of any MHC at least once. We used the following formula to calculate the ICC [34]: ICC = covariance parameter estimate/(covariance parameter estimate + 3.29), and found negligible variance explained by schools (6.5% in girls and 2.1% in boys, see supplementary Table s3). In addition, estimates produced using the three-level hierarchical model were very similar to those of the main analyses (see supplementary Fig. s1 and Table s4).

Because adolescents with no/mild symptoms may have contacted care before follow-up, and eventually improved, we ran the main analysis excluding adolescents who had previously contacted care, 6 months before start of follow-up in 7th, 8th, and 9th grade.

Due to a moderate inter-rater agreement (0.40) between self-rated SDQ and parent-rated SDQ [31], we decided to use parent-reported symptoms as the moderator for comparisons. We categorised adolescents into "no/mild symptoms” for adolescents who scored 0–13, "moderate symptoms” for adolescents who scored 14–16, and “severe symptoms” for those who scored 17–40 on the total difficulties score based on parent reports [19]. Furthermore, due to the large number of missing values on the variable on self-reported mental health status (10%), we replaced the missing with parent-reported values where available per grade. Missing reduced from 10 to 3% with this approach. Using this new variable as the moderator, our supplementary analysis yielded results largely consistent with the main analysis (see supplementary Fig. s2 and Table s5 for comparisons).

Data analysis was performed using SAS statistical software version 9.4 and graphs were produced using RStudio (R version 4.2.2).

## Results

A cohort of 3517 adolescents was followed up from 7 to 9th grade, producing a total of 10,551 observations. Over the follow-up period, adolescents often reported no/mild symptoms (74.8%), moderate symptoms (9.4%), and severe symptoms (5.6%), with the remainder missing. Compared to adolescents with no/mild symptoms, those with moderate-to-severe symptoms were more likely girls, in 9th grade (older), with lower SEP, born to foreign-born parents, with parental mental illness, and they were more likely to utilise MHC (see Table 1). Furthermore, we observed a socioeconomic gradient in adolescents’ mental health status, i.e., a stepwise decrease in the proportion of adolescents with moderate-to-severe symptoms as SEP increased (see supplementary Fig. s3). Girls, 9th-grade adolescents, those with lower SEP, Swedish-born parents, and parents with a history of mental illness were more likely to use MHC (see supplementary Table s6 for grade-specific characteristics). However, among adolescents with moderate-to-severe symptoms, the proportion utilising MHC was higher among those with higher SEP (see Table 1). Finally, except for inpatient care, the use of outpatient care and psychotropic medication was more common among adolescents with lower SEP (*p* value = 0.0001; see supplementary Table s7 for crosstabulations between SEP and MHC use).

**Table 1:** Characteristics of adolescents by frequency (number of observations) of self-reported mental health status and the use of MHC from 7 to 9th grade (each adolescent has up to 3 observations)

			Self-reported mental health status^a^							
	Total		No/mild symptoms		Moderate		Severe		Missing	
Variable	f (col %)	% MHC use	f (row %)	% MHC use	f (row %)	% MHC use	f (row %)	% MHC use	f (row %)	% MHC use
Sex										
Girls	5376 (51.0%)	10.1%	3846 (71.5%)	5.7%	640 (11.9%)	16.1%	420 (7.8%)	28.6%	470 (8.7%)	21.7%
Boys	5175 (49.0%)	8.7%	4050 (78.3%)	6.4%	355 (6.9%)	15.8%	174 (3.4%)	23.6%	596 (11.5%)	15.8%
School grade										
7th	3517 (33.3%)	7.5%	2795 (79.5%)	5.5%	319 (9.1%)	11.0%	161 (4.6%)	21.7%	242 (6.9%)	16.5%
8th	3517 (33.3%)	9.7%	2635 (74.9%)	5.4%	337 (9.6%)	19.3%	202 (5.7%)	33.2%	343 (9.8%)	19.2%
9th	3517 (33.3%)	11.0%	2466 (70.1%)	7.3%	339 (9.6%)	17.4%	231 (6.6%)	25.5%	481 (13.7%)	18.7%
Parent’s education										
Without tertiary education	2975 (28.2%)	11.7%	2021 (67.9%)	7.7%	332 (11.2%)	13.9%	245 (8.2%)	24.5%	377 (12.7%)	22.5%
With tertiary education	7282 (69.0%)	8.7%	5663 (77.8%)	5.5%	633 (8.7%)	17.5%	333 (4.6%)	30.0%	653 (9.0%)	16.5%
Missing	294 (2.8%)	5.1%	212 (72.1%)	4.2%	30 (10.2%)	–	16 (5.4%)	–	36 (12.2%)	–
Household income										
Lowest	3420 (32.4%)	10.2%	2421 (70.8%)	6.2%	370 (10.8%)	15.9%	207 (6.1%)	22.2%	422 (12.3%)	22.3%
Middle	3422 (32.4%)	11.0%	2579 (75.4%)	7.8%	323 (9.4%)	18.0%	202 (5.9%)	30.7%	318 (9.3%)	16.7%
Highest	3422 (32.4%)	7.4%	2692 (78.7%)	4.2%	273 (8.0%)	15.0%	169 (4.9%)	30.8%	288 (8.4%)	16.3%
Missing	287 (2.7%)	4.5%	204 (71.1%)	4.4%	29 (10.1%)	–	16 (5.6%)	–	38 (13.2%)	–
Parent’s country of birth										
At least one born in Sweden	9363 (88.7%)	9.5%	7105 (75.9%)	6.0%	888 (9.5%)	16.7%	518 (5.5%)	27.8%	852 (9.1%)	20.0%
Both or single parent born outside Sweden	939 (8.9%)	8.0%	627 (66.8%)	5.6%	78 (8.3%)	11.5%	64 (6.8%)	21.9%	170 (18.1%)	10.0%
Missing	249 (2.4%)	10.4%	164 (65.9%)	7.3%	29 (11.6%)	–	12 (4.8%)	–	44 (17.7%)	20.5%
Parental mental illness										
Yes	2203 (20.9%)	15.7%	1513 (68.7%)	10.0%	232 (10.5%)	22.0%	165 (7.5%)	30.3%	293 (13.3%)	31.7%
No	8348 (79.1%)	7.7%	6383 (76.5%)	5.1%	763 (9.1%)	14.2%	429 (5.1%)	25.9%	773 (9.3%)	13.3%

(a) Self-reported mental health status was measured at each grade

(-) For data integrity purposes, fewer than 7 cases in a cell are not presented

% MHC use—proportion within a category, e.g., girls who used MHC at least once

f(row%)—frequency and proportion within a category, e.g., how many times girls reported no/mild symptoms

### Moderated association between SEP and the likelihood of utilising MHC at least once

#### Adolescents with no/mild symptoms

**Fig. 2:**
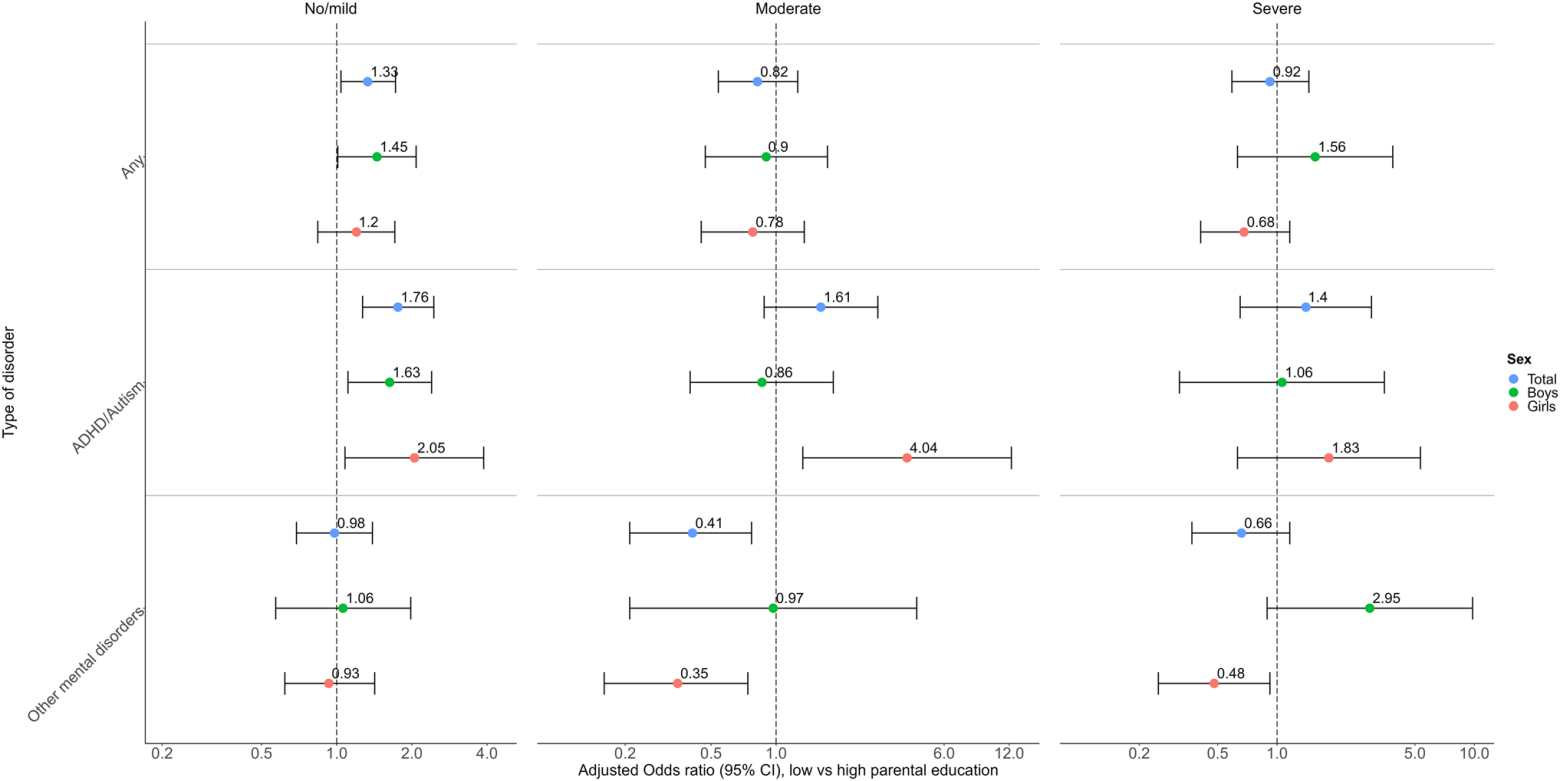
Adjusted odds ratio (log-scale) for the moderated association between parents’ education and utilising MHC (by disorder-group) at least once for 12 months following each survey. Association moderated by adolescents’ self-reported mental health status at each grade. All models were adjusted for calendar year, region of residence, parent’s country of birth, and parental mental illness (and adjusted for sex in the unstratified model, Total). Vertical bars represent 95% CIs

As shown in Fig. 2, lower parental education was significantly associated with higher odds of utilising MHC at least once among adolescents with no/mild symptoms (odds ratio, OR = 1.33 (95% CI 1.04–1.72)). In addition, analysis by type of mental disorder and sex showed that this only applied to boys and girls with ADHD/ASD (see Fig. 2 and supplementary Table s8).

A similar pattern of results was found for the association between income and contact with MHC, especially in boys (see Supplementary Fig. s4 and Table s8).

#### Adolescents with moderate symptoms

There were no statistically significant differences by parental education in the odds of utilising MHC when adolescents reported moderate symptoms (See Fig. 2). However, as shown in Fig. 2, girls to parents without tertiary education had higher odds for ADHD/ASD treatment (OR = 4.04 (95% CI 1.33–12.29), but lower odds of utilising MHC for other mental disorders (OR = 0.35 (95% CI 0.16–0.74)).

Income differences in the odds of utilising MHC were mainly statistically non-significant among adolescents (see supplementary Fig. s4 and Table s8). Sex-stratified analysis showed that boys in households with lower income were approximately two times more likely to utilise MHC than boys in households with the highest income (OR = 1.86 (95% CI 0.90–3.86; non-significant) and OR = 2.34 (95% CI 1.11–4.91) among boys in the lowest and respectively middle income tertiles; see supplementary Table s8).

#### Adolescents with severe symptoms

There were no statistically significant differences by parental education in the odds of utilising MHC among adolescents with severe symptoms (see Fig. 2). Analysis by type of mental disorder and sex showed varying relationships between SEP and MHC use. We found no significant differences in the utilisation of MHC for treating ADHD/ASD but for treating other mental disorders in this group with severe symptoms. In girls, lower parental education predicted lower odds of utilising MHC (OR = 0.48 (95% CI: 0.25 – 0.92)), whereas in boys, lower parental education predicted higher odds of utilising MHC for treating other mental disorders (OR = 2.95 (95% CI: 0.89 – 9.80), but this estimate was not statistically significant.

Lower income was associated with higher odds of using MHC for treating ADHD/ASD but associated with lower odds of utilising MHC for treating other mental disorders mostly among girls, but estimates were non-significant (see supplementary Fig. s4 and Table s8).

#### Results from the sensitivity analysis

A similar pattern of results was found using mental health status reported by parents as the moderator, except for a few differences (see supplementary Fig. s5 and Table s9). For instance, the significant differences by parental education in the odds of utilising MHC for other mental disorders than ADHD/ASD, among girls with moderate-to-severe symptoms, were non-significant (OR = 1.03 (95% CI 0.32–3.29) and respectively OR = 0.49 (95% CI 0.18, 1.32)) when we used parent-reported symptoms instead (see supplementary Table s9).

Results from the analysis that excluded adolescents with previous visits were similar to the main analysis; that is, lower SEP was associated with higher odds of utilising MHC for the treatment of ADHD/ASD among adolescents with no/mild symptoms (see supplementary Fig. s6).

### Moderated association between SEP and the number of outpatient visits

#### Adolescents with no/mild symptoms

**Fig. 3:**
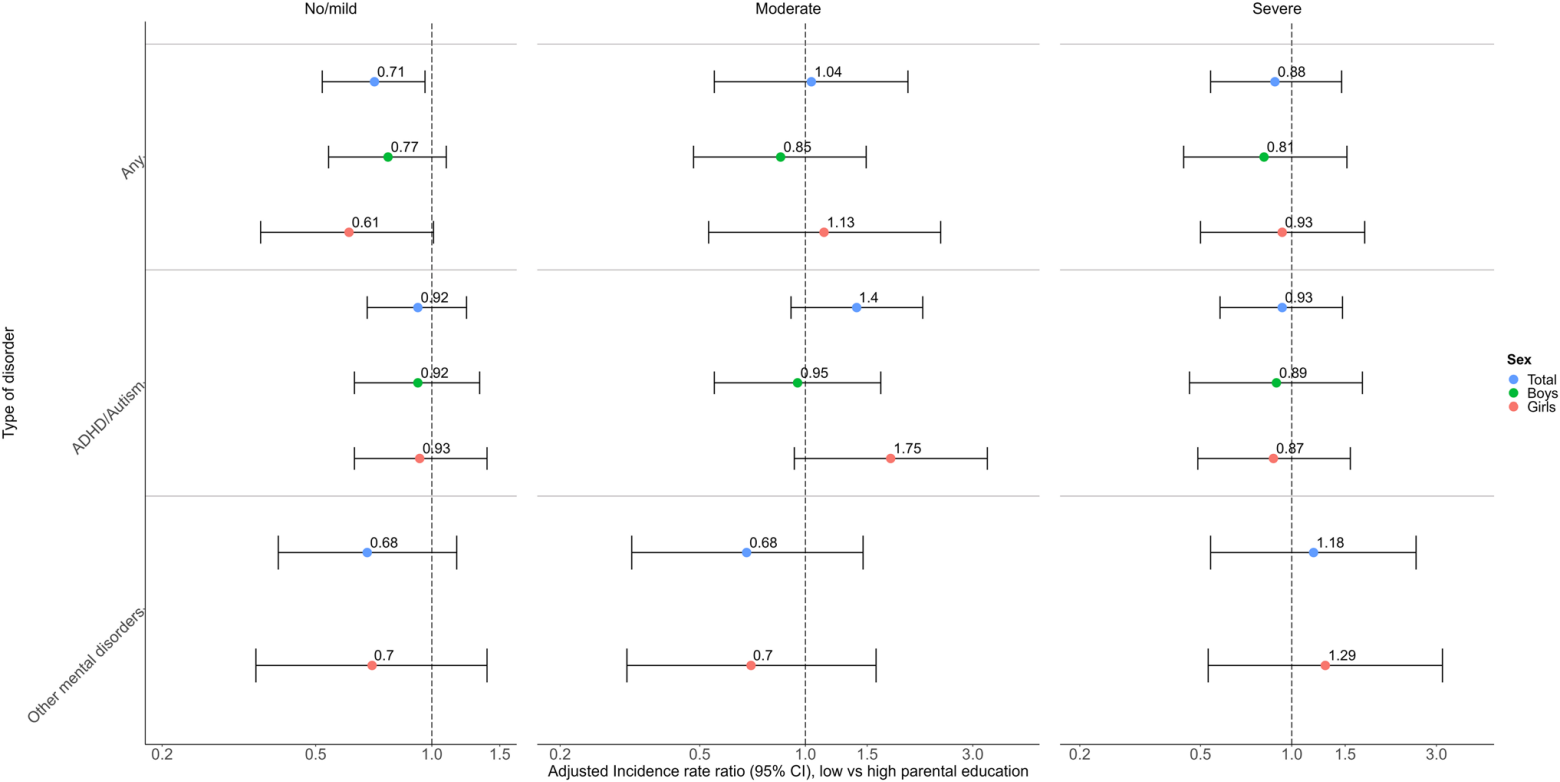
Adjusted incidence rate ratios (log-scale) for the moderated association between parents’ education and number of outpatient visits (by disorder group) during 12 months after each survey. Association moderated by adolescents’ self-reported mental health status at each grade. All models were adjusted for calendar year, region of residence, parent’s country of birth, and parental mental illness (and adjusted for sex in the unstratified model, Total). Vertical bars represent 95% CIs

As shown in Fig. 3, lower parental education was associated with fewer outpatient visits in secondary care when adolescents reported no/milder symptoms (incidence rate ratio, IRR = 0.71 (95% CI 0.52–0.96)). This pattern was largely explained by the utilisation of MHC by girls with other mental disorders (models for other mental disorders did not converge in boys; see Fig. 3). In addition, we found comparable results for the relationship between household income and the use of MHC (lowest versus highest income; IRR = 0.57 (95% CI 0.40–0.80); see supplementary Fig. s7). Results were largely comparable by type of mental disorder and sex (see supplementary Fig. s7 and Table s10).

#### Adolescents with moderate symptoms

There were no statistically significant differences by parental education in the number of outpatient visits when adolescents reported moderate symptoms (see Fig. 3). Results by type of disorder and sex were also non-significant (see Fig. 3). In contrast, lower household income was associated with fewer outpatient visits for any disorder among boys but not girls (IRR = 0.44 (95% CI 0.23–0.86) lowest versus highest income and IRR = 0.44 (95% CI 0.23–0.84) middle versus highest income). Results were comparable for outpatient visits for ADHD/ASD treatment (models for other mental disorders did not converge in boys; see supplementary Fig. s7 and Table s10).

#### Adolescents with severe symptoms

There were no statistically significant differences by parental education in the number of outpatient visits when adolescents reported severe symptoms (see Fig. 3). We found comparable results by type of mental disorder and sex.

There were, however, statistically significant income differences in the number of outpatient visits, particularly among girls. Girls in households with the lowest household income had significantly fewer outpatient visits than their peers (IRR = 0.39 (95% CI 0.19–0.79)). Analysis by type of mental disorder showed similar results for use of outpatient care among girls with other mental disorders, but the estimate was not statistically significant (IRR = 0.51 (95% CI 0.25–1.05); see supplementary Fig. s7 and Table s10).

#### Results from the sensitivity analysis

The results using mental health status reported by parents as the moderator were somewhat different. There were no significant differences by education in the number of outpatient visits when parents reported no/mild symptoms. Furthermore, adolescents to parents with shorter education had fewer outpatient visits compared to other adolescents, when parents reported severe symptoms (IRR = 0.63 (95% CI 0.39–1.01), although statistically non-significant). In addition, among girls with other mental disorders, income differences in the use of outpatient services were only present when parents reported no/mild-to-moderate symptoms but not severe symptoms (See supplementary Table s11).

Excluding adolescents with previous use of any MHC produced comparable results to those of the main analysis among adolescents reporting no/mild symptoms (IRR = 0.64 (95% CI 0.39–1.04) low vs high education).

## Discussion

This study aimed to investigate if there are socioeconomic differences in the utilisation of MHC by Swedish adolescents when accounting for their need for MHC. Our findings suggest that the estimated needs for MHC among adolescents seem to be met equally in Sweden for the treatment of ADHD/ASD. In contrast, there are indications of unequal MHC use among girls with mental disorders such as depression and anxiety.

Consistent with previous research [4, 5], adolescents from families of lower SEP were more likely to suffer from mental health problems compared to other adolescents. This might explain why these adolescents were also more likely to contact MHC than their peers from families of higher SEP. This finding is in agreement with those of other studies from the Nordic countries, where lower SEP has been shown to predict the use of ADHD medication [15, 35] and overall utilisation of inpatient and outpatient secondary MHC [16, 17]. In our study, however, lower SEP mostly predicted higher odds of contacting MHC for ADHD/ASD in adolescents who reported no/mild symptoms. The socioeconomic differences in contacting MHC observed among adolescents with no/mild-to-moderate symptoms could be due to differences in pathways to care or differences in access to protective resources. For instance, on the one hand, there is a possibility that adolescents from families of lower SEP with ADHD/ASD are more likely than their peers to be identified by schools and social services and referred to MHC services [36]. On the other hand, adolescents from families of higher SEP and with milder symptoms may have better access to protective resources such as engagement in leisure activities, social networks, role models, academic support, etc. [37], while those from families of lower SEP may need to rely on support from MHC services. However, once adolescents were in contact with MHC, we found non-significant differences in the frequency of visits, sometimes favouring adolescents with higher SEP, in this group with no/mild symptoms.

We found indications of unequal use of MHC for other mental disorders than ADHD/ASD among girls. The fact that this was apparent for girls with moderate-to-severe symptoms is of great concern, since this might suggest unmet needs among girls from families of lower SEP. Typically, girls may be unrecognised when suffering from depression and anxiety, whereas adolescents, specifically boys with ADHD/ASD, are noticed in the school setting. Perhaps, stigma, inability to recognise symptoms or the need for care, might also explain the observed inequity among girls with mental disorders, such as depression and anxiety [8, 38–40]. Since this finding also concerns the number of visits for those girls who were in contact with MHC, our results may suggest non-compliance with planned care or structural problems in the health care system to support this group of girls with severe symptoms. Availability of services, such as the number of specialists in municipalities, different referral patterns or provider decisions, and waiting time, might impact differently in SEP subgroups [41, 42]. Furthermore, individuals of higher SEP, in this case parents and their children, could be more skilled than others of lower SEP in explaining symptoms, asking for and understanding information, being involved in the decision-making (“better negotiators”), and better at actively requesting appointments with specialists or follow-up visits [41, 42].

In summary, our findings suggest that parental SEP might influence MHC use differently depending on the type of mental health problem. Parents and other actors such as teachers, who interact with adolescents in the community, might collectively play a role in the recognition of MHC needs among adolescents with externalising problems, since these problems are more disruptive in nature, e.g., ADHD. While given the invisible/covert nature of internalising problems (e.g., depression and anxiety), the role of recognising symptoms might solely fall on adolescents or their parents, and this ability to recognise symptoms could differ by SEP.

### Strengths and limitations

Our study has potential limitations. First, we did not have a comprehensive measure of need such as adolescents’ perceived need for services and clinically assessed need. However, our choice of indicator for need for MHC, mental health status measured using the SDQ scales, has been shown to discriminate a clinical population from controls and to predict care seeking well [31]. Second, we did not have information on adolescents’ utilisation of school health services and primary care, where milder forms of mental health problems might be treated, and this might differ between SEP subgroups. However, adolescents in Sweden can access specialist services directly without referrals from primary care [43] and the large majority of adolescents are treated (defined as recorded mental disorder diagnoses) in secondary care as compared to primary care [44]. In addition, we had data on receipt of prescriptions from both primary and secondary care, and hence, we might have captured a sizeable number of those treated in primary care only. Third, because of selection processes at inception, the Kupol cohort comprises a large number of participants from families of higher SEP [19], and may not be representative of the general population of adolescents in Sweden. However, such a selection would presumably result in an underestimation rather than an overestimation of differences by SEP.

Our study has major strengths: we used a fairly large sample size, data were collected yearly, and we had access to subjectively measured mental health status (both self-reported and parent-reported), and objectively measured SEP indicators and MHC use from administrative registries.

### Future research and implications of our findings

Future research should expand on our findings using other indicators of need, preferably self-perceived and provider-assessed need. In addition, research should examine potential barriers, and compliance to MHC among girls with depression and anxiety, from families of lower SEP. Both user and provider perspectives could provide rich insights to understand how to meet the needs of these adolescents.

## Conclusion

In this sample of Swedish adolescents, the use of MHC for ADHD/ASD treatment was influenced by needs, whereas we found indications of unequal use of MHC for the treatment of other mental disorders among girls with self-reported moderate-to-severe symptoms. Patterns to use and retention in secondary outpatient care for girls from families of lower SEP warrant further attention.

### Supplementary Information

Below is the link to the electronic supplementary material.

Supplementary file1 (PDF 1276 KB)

## Data Availability

Requests for access to data should be sent to MRG.
