# Supplementary material for: Are the estimated needs for mental health care among adolescents from different socioeconomic backgrounds met equally in Sweden? A longitudinal survey-registry linkage study
Source: Eur Child Adolesc Psychiatry. 2023 Dec 29;33(8):2581–91. doi: 10.1007/s00787-023-02341-2 (PMC11272712; doi:10.1007/s00787-023-02341-2)
Supplement: Supplementary file 1 — Supplementary file1 (PDF 1276 KB) [file 787_2023_2341_MOESM1_ESM.pdf]

Title: Are the estimated needs for mental health care among adolescents from different socioeconomic backgrounds met equally in Sweden? A longitudinal survey-registry linkage study.

Joseph Jr. Muwonge MSc[1,2] (ORCID: 0000-0002-9219-9752), Prof Christina Dalman PhD[1,2] (ORCID: 0000-0002-3579-2357), Prof Bo Burström PhD[1,2] (ORCID: 0000-0001-5770-9422), Antonio Ponce de Leon PhD[1,2], Prof Maria Rosaria Galanti PhD[2] (ORCID: 0000-0002-7805-280X), Beata Jablonska PhD[1,2] (ORCID: 0000-0002-0246-6643), Associate Prof Anna-Clara Hollander PhD[2] (ORCID:0000-0002-1246-5804)

1. Centre for Epidemiology and Community Medicine, Stockholm, Sweden
2. Department of Global Public Health, Karolinska Institute, Stockholm, Sweden

Corresponding author

Name: Joseph Jr. Muwonge

Contact: joseph.junior.muwonge@ki.se

Funding: The original research project "Kupol longitudinal study" was funded by the Swedish Research Council for sustainable development, FORMAS; the Swedish Research Council for Health, Working Life and Welfare, FORTE; and The Swedish Research council.

Contents:

Supplementary Figures

1. Fig s1. Comparison of estimates produced using proc Genmod (GEE models – Model1) and proc Glimmix (hierarchical generalized linear models- Model2).
2. Fig s2. Comparison of estimates produced from the main analysis using complete cases (model1) and that using self-reported mental health status and parent reported when missing/imputed (model2).
3. Fig s3. Distribution of Adolescents' self-reported mental health status by SEP, data from 7th to 9th grade.
4. Fig s4. Adjusted odds ratio for the moderated association between household income and utilising any mental healthcare service at least once for 12 months following each survey.
5. Fig s5. Comparison of estimates produced from the main analysis using self-reported mental health status (model1) and that using parent reported mental health status (model2).
6. Fig s6. Association between SEP and utilisation of any MHC at least once, excluding adolescents who utilised MHC in the previous 6 months before follow-up (i.e., incident utilisation)
7. Fig s7. Adjusted incidence rate ratio for the moderated association between household income and number of outpatient visits for 12 months following each survey.

Supplementary Tables

1. Table s1. ICD-10 codes, DSM IV codes, and ATC codes used to capture use of mental healthcare services.
2. Table s2. Syntax for analysis of the models predicting utilising MHC at least once.
3. Table s3. Intra class coefficients (ICC) for variance explained by schools in utilisation of any MHC at least once.
4. Table s4. Comparison of estimates between proc glimmix (hierarchical generalized linear models) and proc genmod (GEE models). Adjusted odds ratio for association between SEP and utilisation of any MHC at least once by adolescents.
5. Table s5. Comparison of estimates between only self-reported mental health status as moderator and imputed version as moderator (self-reported, where missing replaced with parent reported mental health status).
6. Table s6. Characteristics of adolescents according to any contact with mental healthcare services from 7th (baseline) to 9th grade (follow up).
7. Table s7. Distribution of utilisation by level/type of care and socioeconomic position for all 3 years of follow-up.
8. Table s8. Association between SEP and utilisation of any MHC (from 7th to 9th grade), moderated by adolescents' self-reported mental health status status.
9. Table s9. Association between SEP and utilisation of any MHC (from 7th to 9th grade), moderated by adolescents' parent-reported mental health status.
10. Table s10. Association between SEP and number of outpatient visits to secondary care (from 7th to 9th grade), moderated by adolescents' self-reported mental health status.
11. Table s11. Association between SEP and number of outpatient visits to secondary care (from 7th to 9th grade)

Table s1. ICD-10 codes, DSM IV codes, and ATC codes used to capture use of mental healthcare services

Type	Codes	source	Notes
Any Mental health care (MHC) use	F0-F99, G47, X60-X84, Z91.5, R45, Z72.820, Z73.3, Z73.4, Z73.9, Z72.810, Z032.<br><br>+ all visits within Child and Adolescent Psychiatry clinics (CAP)<br><br>ATC: N05A, N05B, N05C, N06A, and N06B	Inpatient, and outpatient records from the National Patient Register (NPR) + regional CAP registers + Prescribed Drugs register	*The NPR contains all care provided by doctors in publicly funded secondary health care services.*<br><br>*Regional CAP registers contain all visits regardless of healthcare provider met.*<br><br>*The Prescribed Drugs register contains all prescriptions collected.*<br><br>*We have considered that all visits to CAP are due to mental health problems and have included all visits in in the main outcome.*<br><br>*To capture all potential forms of utilisation of mental health care, we included treatment of behaviours and disorders outside chapter F. For instance, sleep problems/disorders often present as early symptoms of emotional and behavioural problems in children and suicidal behaviour might represent serious emotional problems in adolescents (and cause for hospitalisation in psychiatric clinics).*
MHC use for neuropsychiatric conditions (ADHD/Autism)	ICD-10: F84, F90, DSM-IV: 314, 312, 299,<br><br>ATC: N06B	Same as above	Only relevant visits (registered ADHD/ASD diagnosis) in regional CAP registers and the NPR were retrieved.
MHC use for other mental disorders	All codes excluding care for neuropsychiatric conditions.	Same as above	

Table s2. Syntax for analysis of the models predicting utilising MHC at least once

Syntax/analysis code for the first question: Socioeconomic differences in use of MHC at least once for 12 months following survey response

```
proc genmod data=long_dataset;
by sex; /*stratification by sex*/
class mentalhealth_status studentID Tertiary_education(ref="1"); /*reference category is parents with tertiary education*/
model Any_mentalhealthcare (event='1')= mentalhealth_status
Tertiary_education(mentalhealth_status) calendar_year region_of_residence
Parental_countryofbirth parental_mentalillness / dist=bin maxiter=100;  /*increasing possible interations for model convergence */
repeated subject=studentID / type=exch corrw; /*exch- exchangeable correlation structure, corrw tells SAS to print the correlation matrix*/
ods output GEEEmpPEst = mycoefficients;    /*betas and their confidence intervals are output in a table for transformation into odds ratios*/
run;
/*transforming betas to odds ratios and 95% CIs*/
data MyORs;
set mycoefficients;
OR= exp(estimate);
Low_CI = exp(LowerCL);
High_CI = exp(UpperCL);
if parm ^="Intercept";
drop Estimate LowerCL UpperCL StdErr Z; run;
```

Table s3. Intra class coefficients (ICC) for variance explained by schools in utilisation of any MHC at least once

**Sex**	**ICC**	**P-value**
Girls	6.5%	0.0119
Boys	2.1%	0.2240

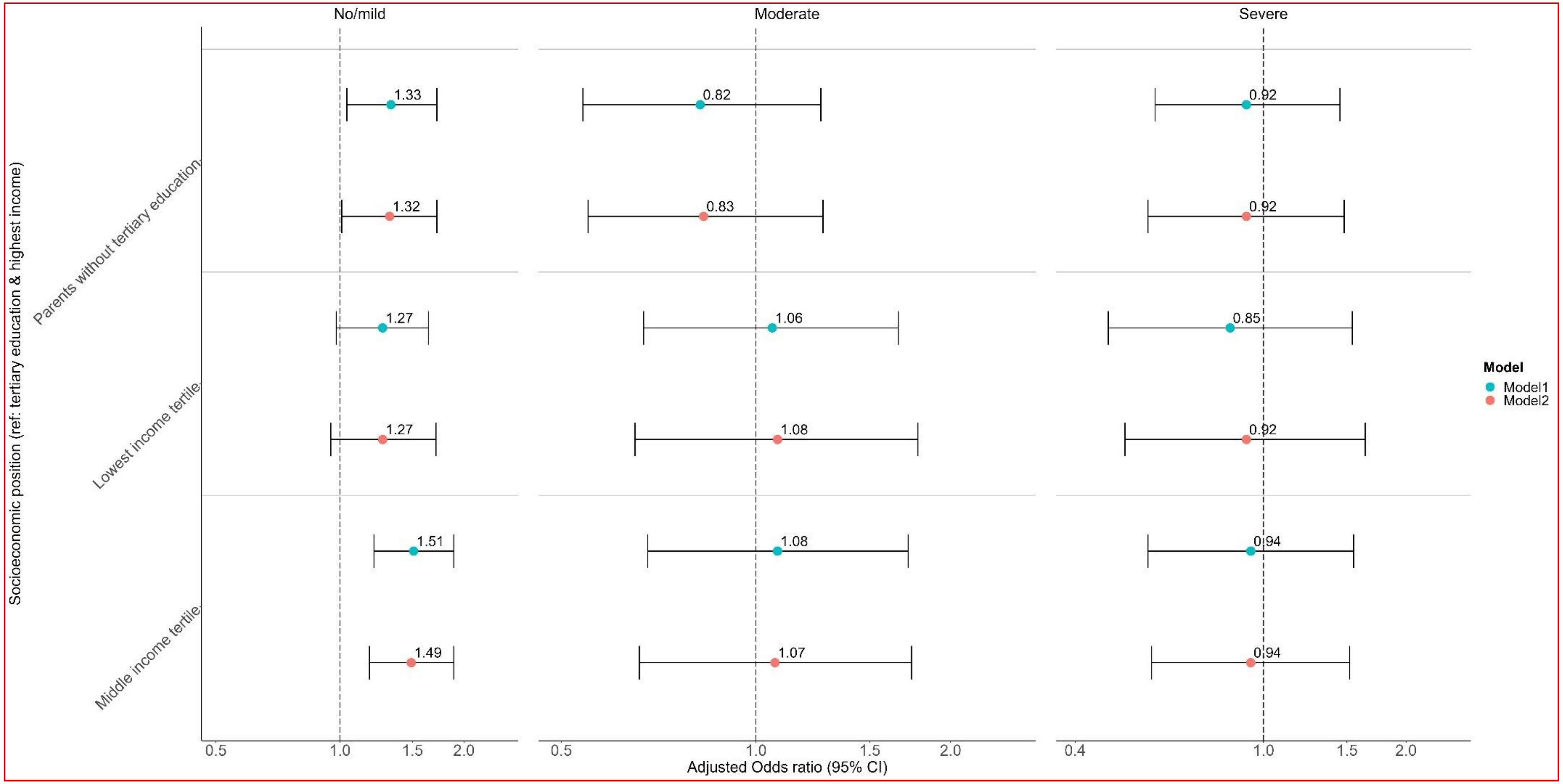

Fig s1. Comparison of estimates produced using proc Genmod (GEE models – Model1) and proc Glimmix (hierarchical generalized linear models- Model2). *Odds ratios (log-scale) for the association between SEP and MHC use. Association moderated by adolescents' self-reported mental health status at each grade. All models were adjusted for calendar year, region of residence, parent's country of birth, parental mental illness, and sex. Vertical bars represent 95% CIs.*

Table s4. Comparison of estimates between proc glimmix (hierarchical generalized linear models) and proc genmod (GEE models). Adjusted odds ratio for association between SEP and utilisation of any MHC at least once by adolescents. Moderated by adolescents' self-reported mental health status.

	SEP	SEP level	proc genmod All ORs (95% CI)	proc glimmix All ORs (95% CI)	proc genmod No/mild symptoms ORs (95% CI)	proc glimmix No/mild symptoms ORs (95% CI)	proc genmod Moderate ORs (95% CI)	proc glimmix Moderate ORs (95% CI)	proc genmod Severe ORs (95% CI)	proc glimmix Severe ORs (95% CI)
**Total**										
Any mental healthcare service	Tertiary education	No	1.13 (0.90, 1.42)	1.18 (0.95, 1.47)*	**1.33 (1.04, 1.72)**	**1.32 (1.01, 1.72)**	0.82 (0.54, 1.26)	0.83 (0.55, 1.27)	0.92 (0.59, 1.45)	0.92 (0.57, 1.48)
	Household income	Low	1.15 (0.91, 1.44)	1.23 (0.97, 1.57)*	1.27 (0.98, 1.64)*	1.27 (0.95, 1.71)*	1.06 (0.67, 1.66)	1.08 (0.65, 1.78)	0.85 (0.47, 1.54)	0.92 (0.51, 1.64)
		Middle	**1.32 (1.07, 1.62)**	**1.28 (1.05, 1.58)**	**1.51 (1.21, 1.89)**	**1.49 (1.18, 1.89)**	1.08 (0.68, 1.72)	1.07 (0.66, 1.74)	0.94 (0.57, 1.55)	0.94 (0.58, 1.52)
**Girls**										
Any mental healthcare service	Tertiary education	No	0.93 (0.68, 1.25)	0.93 (0.70, 1.23)	1.20 (0.84, 1.71)	1.20 (0.86, 1.69)	0.78 (0.45, 1.35)	0.78 (0.46, 1.32)	0.68 (0.41, 1.16)	0.69 (0.40, 1.19)
	Household income	Low	0.85 (0.62, 1.17)	0.88 (0.62, 1.24)	0.93 (0.62, 1.37)	0.96 (0.63, 1.45)	0.83 (0.48, 1.43)	0.85 (0.47, 1.53)	0.67 (0.35, 1.30)	0.72 (0.37, 1.40)
		Middle	1.05 (0.79, 1.39)	1.07 (0.79, 1.43)	1.31 (0.94, 1.82)	1.35 (0.95, 1.92)*	0.74 (0.42, 1.30)	0.75 (0.44, 1.28)	0.78 (0.44, 1.40)	0.78 (0.45, 1.35)
**Boys**										
Any mental healthcare service	Tertiary education	No	1.37 (0.98, 1.92)*	1.35 (0.94, 1.94)	**1.45 (1.01, 2.08)**	1.42 (0.96, 2.12)*	0.90 (0.47, 1.73)	0.91 (0.49, 1.72)	1.56 (0.63, 3.86)	1.51 (0.59, 3.88)
	Household income	Low	**1.63 (1.20, 2.22)**	**1.57 (1.14, 2.17)**	**1.68 (1.22, 2.31)**	**1.61 (1.17, 2.21)**	1.86 (0.90, 3.86)	1.77 (0.80, 3.90)	1.18 (0.37, 3.72)	1.18 (0.35, 3.97)
		Middle	**1.73 (1.31, 2.28)**	**1.63 (1.25, 2.14)**	**1.68 (1.26, 2.23)**	**1.57 (1.19, 2.08)**	**2.34 (1.11, 4.91)**	**2.18 (1.10, 4.30)**	1.62 (0.63, 4.16)	1.66 (0.68, 4.05)

- Reference categories are yes for parents with tertiary education & the highest income tertile.
- Different models run for each SEP indicator.
- Each model was adjusted for calendar year, region of residence, parental country of birth, & parental history of mental illness (and adjusted for sex for the Total model).
- (a) Estimates under All, represent the association between SEP and utilisation of any MHC at least once for all adolescents regardless of their mental health status (adjusted for mental health status + other covariates)
- Statistically significant estimates highlighted in bold font & * represents borderline significance.
- SEP = Socioeconomic position, MHC = Mental health care; OR=Odds ratio

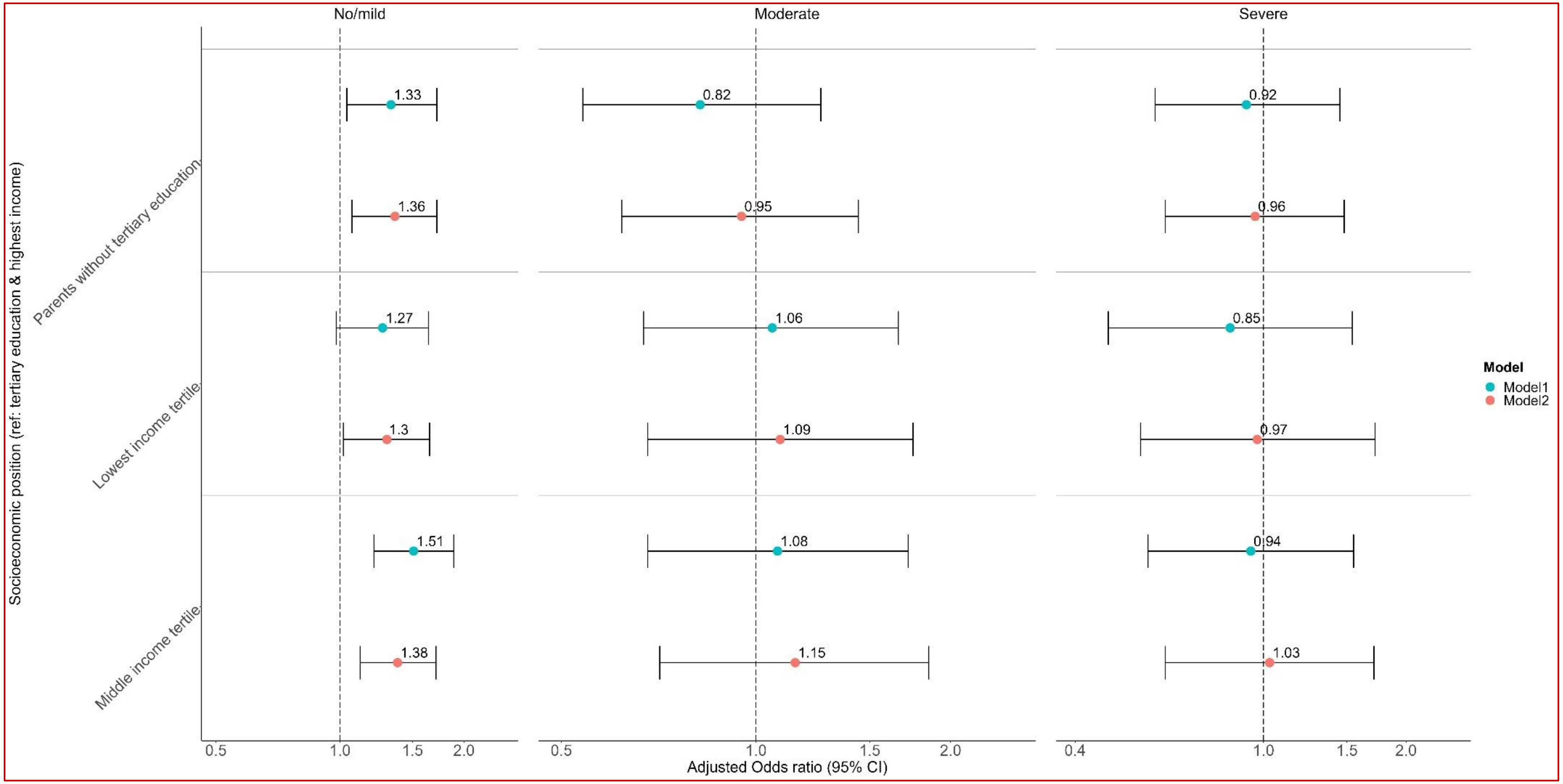

Fig s2. Comparison of estimates produced from the main analysis using complete cases (model1) and that using self-reported mental health status and parent reported when missing/imputed (model2). *Odds ratios (log-scale) for the association between SEP and MHC use. All models were adjusted for calendar year, region of residence, parent's country of birth, parental mental illness, and sex. Vertical bars represent 95% CIs.*

Table s5. Comparison of estimates between only self-reported mental health status as moderator and the imputed version as moderator (self-reported, where missing replaced with parent reported mental health status).

			Only Self-reported SDQ	Self-reported SDQ (imputation with parent SDQ/grade)	Only Self-reported SDQ	Self-reported SDQ (imputation with parent SDQ/grade)	Only Self-reported SDQ	Self-reported SDQ (imputation with parent SDQ/grade)
			No/mild symptoms	No/mild symptoms	Moderate	Moderate	Severe	Severe
			**OR (95% CI)**	**OR (95% CI)**	**OR (95% CI)**	**OR (95% CI)**	**OR (95% CI)**	**OR (95% CI)**
Any MHC use	Tertiary education	No	**1.33 (1.04, 1.72)**	**1.36 (1.07, 1.72)**	0.82 (0.54, 1.26)	0.95 (0.62, 1.44)	0.92 (0.59, 1.45)	0.96 (0.62, 1.48)
	Household income	Low	1.27 (0.98, 1.64)	**1.30 (1.02, 1.65)**	1.06 (0.67, 1.66)	1.09 (0.68, 1.75)	0.85 (0.47, 1.54)	0.97 (0.55, 1.72)
		Middle	**1.51 (1.21, 1.89)**	**1.38 (1.12, 1.71)**	1.08 (0.68, 1.72)	1.15 (0.71, 1.85)	0.94 (0.57, 1.55)	1.03 (0.62, 1.71)
MHC use for Neuropsychiatric conditions	Tertiary education	No	**1.76 (1.27, 2.45)**	**1.84 (1.35, 2.51)**	1.61 (0.88, 2.96)	1.79 (1.00, 3.19)*	1.40 (0.65, 3.01)	1.35 (0.65, 2.79)
	Household income	Low	**1.46 (1.11, 1.90)**	**1.42 (1.10, 1.84)**	1.33 (0.65, 2.70)	1.54 (0.73, 3.26)	1.38 (0.47, 4.09)	1.56 (0.60, 4.02)
		Middle	**1.47 (1.18, 1.84)**	**1.43 (1.14, 1.78)**	1.37 (0.65, 2.89)	1.66 (0.77, 3.57)	1.66 (0.65, 4.21)	1.51 (0.63, 3.62)
MHC use for other mental disorders	Tertiary education	No	0.98 (0.69, 1.39)	**1.00 (0.72, 1.38)**	**0.41 (0.21, 0.77)**	**0.45 (0.24, 0.83)**	0.66 (0.37, 1.16)	0.67 (0.38, 1.18)
	Household income	Low	1.05 (0.72, 1.55)	1.15 (0.81, 1.62)	0.77 (0.43, 1.38)	0.75 (0.42, 1.36)	0.54 (0.27, 1.08)	0.61 (0.31, 1.19)
		Middle	1.36 (0.97, 1.89)	1.23 (0.90, 1.67)	0.78 (0.43, 1.39)	0.77 (0.42, 1.39)	0.66 (0.36, 1.20)	0.74 (0.40, 1.35)

- Reference categories are yes for parents with tertiary education & the highest income tertile.
- Different models run for each SEP indicator.
- Each model was adjusted for calendar year, region of residence, parental country of birth, parental history of mental illness and sex.
- Statistically significant estimates highlighted in bold font & * represents borderline significance.
- SEP = Socioeconomic position, MHC = Mental health care; OR=Odds ratio

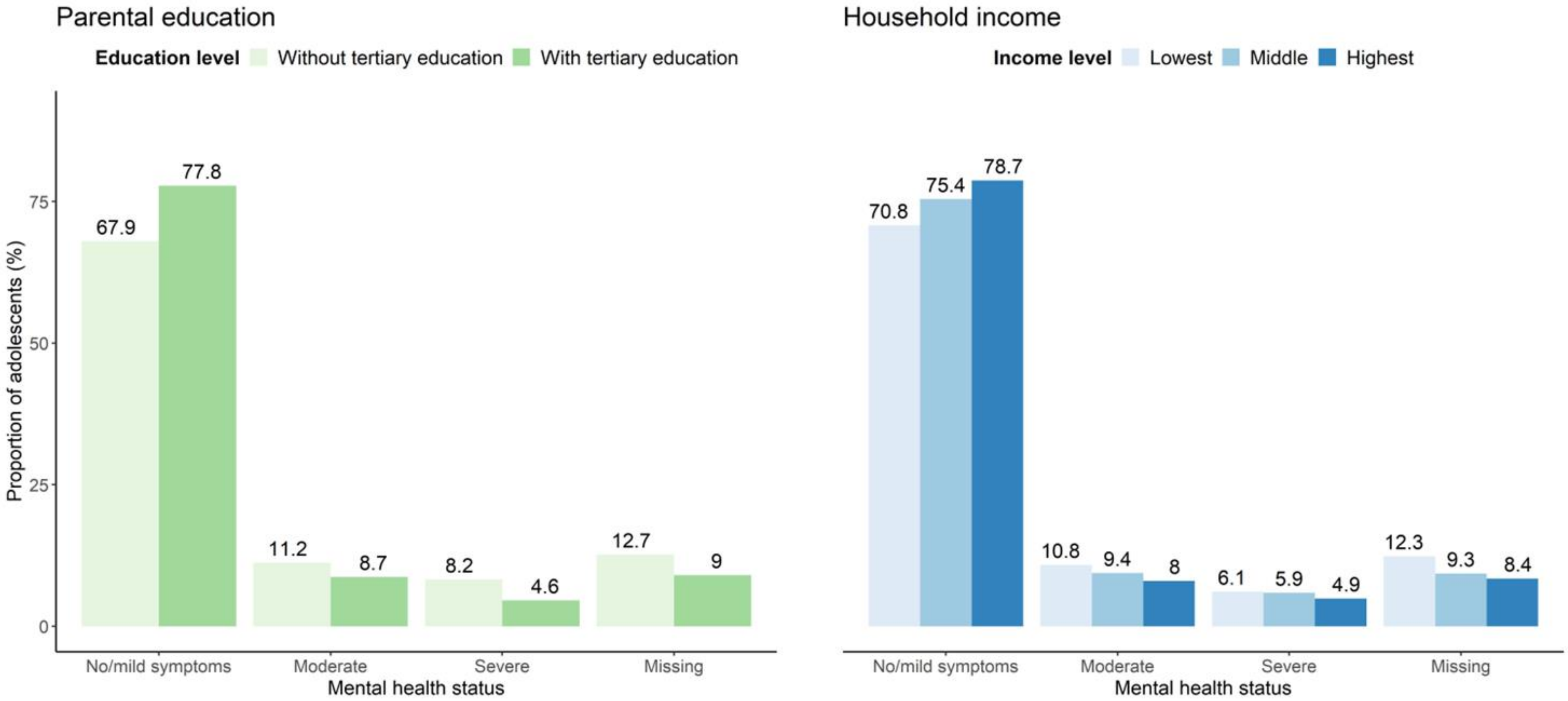

Fig s3. Distribution of Adolescents' self-reported mental health status by SEP, data from 7th to 9th grade. *Proportion of adolescents within SEP categories with no/mild symptoms to severe symptoms.*

Table s6. Characteristics of adolescents according to any contact with mental healthcare services from 7th (baseline) to 9th grade (follow up). *Number and row percent given.*

		**7th grade**		**8th grade**		**9th grade**	
		Total	*≥1 visit to MHC*	*Total*	*≥1 visit to MHC*	*Total*	*≥1 visit to MHC*
		**N**	**n (%)**	**N**	**n (%)**	**N**	**n (%)**
Sex	Girls	1792	129 (7.2%)	1792	191 (10.7%)	1792	223 (12.4%)
	Boys	1725	135 (7.8%)	1725	150 (8.7%)	1725	164 (9.5%)
Parent's education	Without tertiary education	1000	95 (9.5%)	992	123 (12.4%)	983	129 (13.1%)
	With tertiary education	2419	167 (6.9%)	2427	211 (8.7%)	2436	252 (10.3%)
	Missing	98	2 (2.0%)	98	7 (7.1%)	98	6 (6.1%)
Household income	Lowest	1140	91 (8.0%)	1141	116 (10.2%)	1139	143 (12.6%)
	Middle	1142	104 (9.1%)	1140	125 (11.0%)	1140	146 (12.8%)
	Highest	1141	67 (5.9%)	1141	94 (8.2%)	1140	93 (8.2%)
	Missing	94	2 (2.1%)	95	6 (6.3%)	98	5 (5.1%)
Parent's country of birth	At least one born in Sweden	3121	234 (7.5%)	3121	304 (9.7%)	3121	353 (11.3%)
	Both or single parent born outside Sweden	269	23 (7.3%)	269	28 (8.9%)	269	24 (7.7%)
	Missing	83	7 (8.4%)	83	9 (10.8%)	83	10 (12.0%)
Parental mental illness	Yes	688	91 (13.2%)	736	115 (15.6%)	779	140 (18.0%)
	No	2829	173 (6.1%)	2781	226 (8.1%)	2738	247 (9.0%)

MHC – Mental Health Care use measured for 12 months of follow-up at each grade; N-Total number

Table s7. Distribution of utilisation by level/type of care and socioeconomic position for all 3 years of follow-up.

	**Tertiary education**		**P-value**	**Household income**			**P-value**
	**No**	**Yes**		**Lowest**	**Middle**	**Highest**	
**Outpatient care#**			<0.0001				<0.0001
None	2666 (89.6%)	6730 (92.4%)		3111 (91.0%)	3092 (90.4%)	3198 (93.5%)	
At least once	309 (10.4%)	552 (7.6%)		309 (9.0%)	330 (9.6%)	224 (6.5%)	
**Inpatient care**			0.7905				0.4678
None	2963 (99.7%)	7250 (99.6%)		3407 (99.6%)	3407 (99.6%)	3413 (99.7%)	
At least once	10 (0.3%)	27 (0.4%)		13 (0.4%)	15 (0.4%)	9 (0.3%)	
**Medication use**			<0.0001				<0.0001
None	2739 (92.1%)	6869 (94.4%)		3199 (93.5%)	3158 (92.3%)	3265 (95.4%)	
At least once	234 (7.9%)	408 (5.6%)		221 (6.5%)	264 (7.7%)	157 (4.6%)	
	# outpatient care records from the national patient register + visits to Child and Adolescent Psychiatry (CAP)<br>P-value produced from Chi-square tests for differences in groups						

Table s8. Association between SEP and utilisation of any MHC (from 7th to 9th grade), moderated by adolescents' self-reported mental health status. *Adjusted odds ratios with 95% confidence intervals given.*

			All[a]	No/mild symptoms	Moderate	Severe
**Type of service**	**SEP**	**SEP level**	**OR (95% CI)**	**OR (95% CI)**	**OR (95% CI)**	**OR (95% CI)**
**Total (girls and boys)**						
Any MHC use	Tertiary education	No	1.13 (0.90, 1.42)	**1.33 (1.04, 1.72)**	0.82 (0.54, 1.26)	0.92 (0.59, 1.45)
		Yes (ref)	1	1	1	1
	Household income	Low	1.15 (0.91, 1.44)	1.27 (0.98, 1.64)*	1.06 (0.67, 1.66)	0.85 (0.47, 1.54)
		Middle	**1.32 (1.07, 1.62)**	**1.51 (1.21, 1.89)**	1.08 (0.68, 1.72)	0.94 (0.57, 1.55)
		Highest (ref)	1	1	1	1
MHC use for Neuropsychiatric conditions	Tertiary education	No	**1.65 (1.21, 2.26)**	**1.76 (1.27, 2.45)**	1.61 (0.88, 2.96)	1.40 (0.65, 3.01)
		Yes (ref)	1	1	1	1
	Household income	Low	**1.42 (1.08, 1.87)**	**1.46 (1.11, 1.90)**	1.33 (0.65, 2.70)	1.38 (0.47, 4.09)
		Middle	**1.49 (1.18, 1.88)**	**1.47 (1.18, 1.84)**	1.37 (0.65, 2.89)	1.66 (0.65, 4.21)
		Highest (ref)	1	1	1	1
MHC use for other mental disorders	Tertiary education	No	0.76 (0.56, 1.01)*	0.98 (0.69, 1.39)	**0.41 (0.21, 0.77)**	0.66 (0.37, 1.16)
		Yes (ref)	**1**			
	Household income	Low	0.87 (0.63, 1.21)	1.05 (0.72, 1.55)	0.77 (0.43, 1.38)	0.54 (0.27, 1.08)
		Middle	1.07 (0.81, 1.41)	1.36 (0.97, 1.89)*	0.78 (0.43, 1.39)	0.66 (0.36, 1.20)
		Highest (ref)	1	1	1	1
**Girls**						
Any MHC use	Tertiary education	No	0.93 (0.68, 1.25)	1.20 (0.84, 1.71)	0.78 (0.45, 1.35)	0.68 (0.41, 1.16)
		Yes (ref)	1	1	1	1
	Household income	Low	0.85 (0.62, 1.17)	0.93 (0.62, 1.37)	0.83 (0.48, 1.43)	0.67 (0.35, 1.30)
		Middle	1.05 (0.79, 1.39)	1.31 (0.94, 1.82)	0.74 (0.42, 1.30)	0.78 (0.44, 1.40)
		Highest (ref)	1	1	1	1
MHC use for Neuropsychiatric conditions	Tertiary education	No	**2.18 (1.25, 3.78)**	**2.05 (1.08, 3.88)**	**4.04 (1.33, 12.29)**	1.83 (0.63, 5.33)

			All[a]	No/mild symptoms	Moderate	Severe
**Type of service**	**SEP**	**SEP level**	**OR (95% CI)**	**OR (95% CI)**	**OR (95% CI)**	**OR (95% CI)**
		Yes (ref)	1	1	1	1
	Household income	Low	0.94 (0.56, 1.58)	0.80 (0.47, 1.39)	0.90 (0.33, 2.50)	1.29 (0.35, 4.71)
		Middle	1.07 (0.71, 1.62)	1.04 (0.65, 1.66)	0.61 (0.17, 2.25)	1.53 (0.45, 5.15)
		Highest (ref)	1	1	1	1
MHC use for other mental disorders	Tertiary education	No	**0.63 (0.45, 0.89)**	0.93 (0.62, 1.42)	**0.35 (0.16, 0.74)**	**0.48 (0.25, 0.92)**
		Yes (ref)	1	1	1	1
	Household income	Low	0.81 (0.56, 1.18)	0.94 (0.59, 1.51)	0.78 (0.41, 1.48)	0.54 (0.25, 1.17)
		Middle	0.98 (0.71, 1.36)	1.31 (0.89, 1.93)	0.74 (0.39, 1.40)	0.56 (0.28, 1.13)
		Highest (ref)	1	1	1	1
**Boys**						
Any MHC use	Tertiary education	No	1.37 (0.98, 1.92)	**1.45 (1.01, 2.08)**	0.90 (0.47, 1.73)	1.56 (0.63, 3.86)
		Yes (ref)	1	1	1	1
	Household income	Low	**1.63 (1.20, 2.22)**	**1.68 (1.22, 2.31)**	1.86 (0.90, 3.86)	1.18 (0.37, 3.72)
		Middle	**1.73 (1.31, 2.28)**	**1.68 (1.26, 2.23)**	**2.34 (1.11, 4.91)**	1.62 (0.63, 4.16)
		Highest (ref)	1	1	1	1
MHC use for Neuropsychiatric conditions	Tertiary education	No	1.40 (0.96, 2.04)*	**1.63 (1.11, 2.40)**	0.86 (0.40, 1.84)	1.06 (0.32, 3.50)
		Yes (ref)	1	1	1	1
	Household income	Low	**1.71 (1.27, 2.31)**	**1.79 (1.34, 2.40)**	1.83 (0.73, 4.54)	1.10 (0.21, 5.83)
		Middle	**1.70 (1.30, 2.24)**	**1.65 (1.30, 2.10)**	2.27 (0.90, 5.75)	1.53 (0.39, 5.97)
		Highest (ref)	1	1	1	1
MHC use for other mental disorders	Tertiary education	No	1.24 (0.72, 2.14)	1.06 (0.57, 1.98)	0.97 (0.21, 4.48)	2.95 (0.89, 9.80)
		Yes (ref)	1	1	1	1
	Household income	Low	1.20 (0.65, 2.23)	1.31 (0.68, 2.53)	1.32 (0.16, 10.85)	0.68 (0.12, 3.67)
		Middle	1.43 (0.82, 2.48)	1.46 (0.78, 2.71)	1.83 (0.24, 13.99)	1.22 (0.33, 4.58)
		Highest (ref)	1	1	1	1

			All[a]	No/mild symptoms	Moderate	Severe
Type of service	SEP	SEP level	OR (95% CI)	OR (95% CI)	OR (95% CI)	OR (95% CI)

- Reference categories are yes for parents with tertiary education & the highest household income tertile
- Different models were run for each SEP indicator
- Each model was adjusted for calendar year, region of residence, parent's country of birth, & parental mental illness (and adjusted for sex for the Total model)
- (a) Estimates under all, represent the association between SEP and at least one visit for all adolescents regardless of their mental health status (adjusted for mental health status + other covariates)
- Statistically significant estimates highlighted in bold font
- * Borderline significance

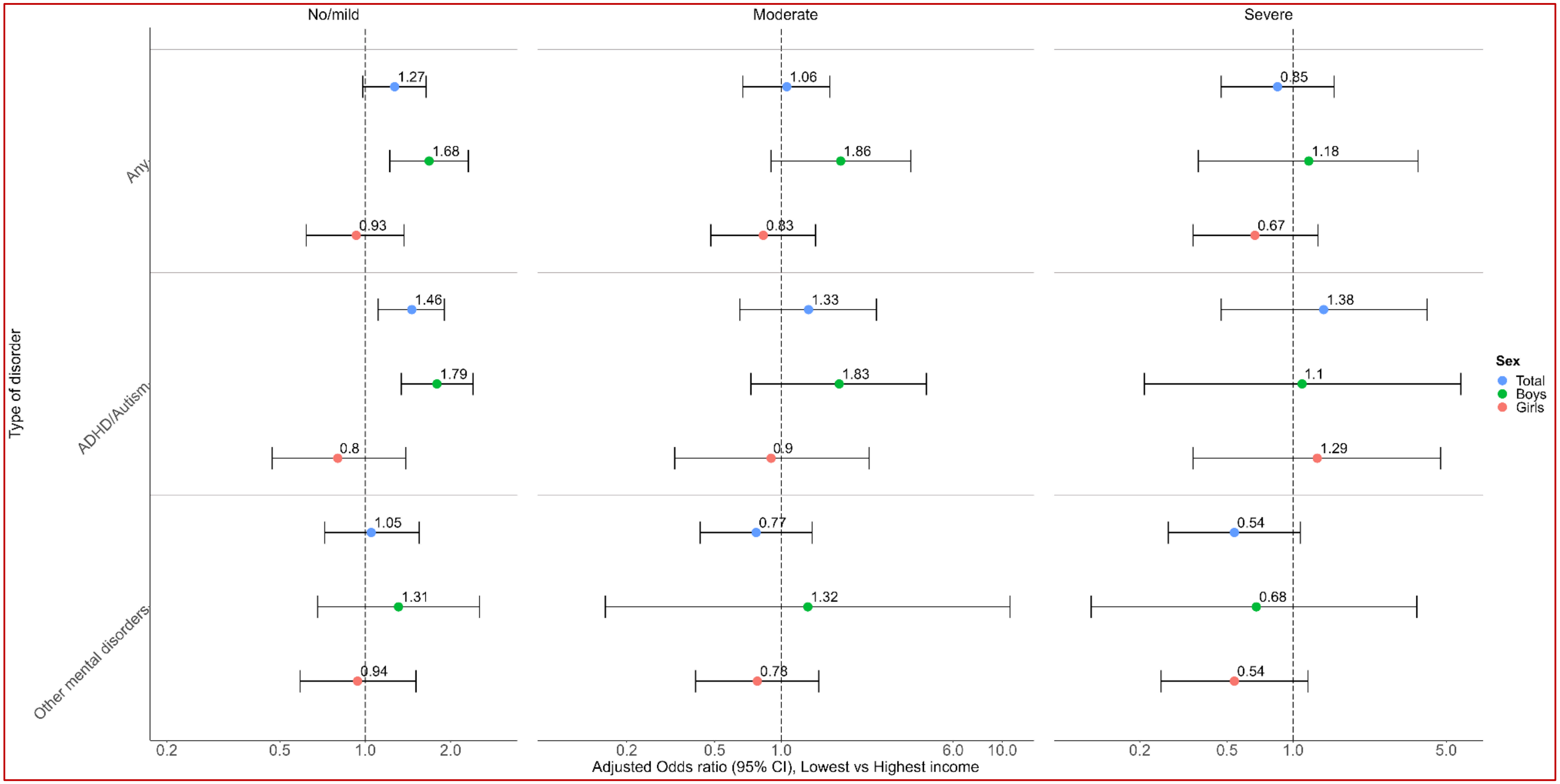

Fig s4. Adjusted odds ratios (log-scale) for the moderated association between household income and utilising any mental healthcare service at least once for 12 months following each survey. *Association moderated by adolescents' self-reported mental health status at each grade. All models were adjusted for calendar year, region of residence, parent's country of birth, parental mental illness (and adjusted for sex in the unstratified model, Total). Vertical bars represent 95% CIs. Note: lowest tertile vs Highest tertile, see table s8 for comparison between middle tertile and highest tertile.*

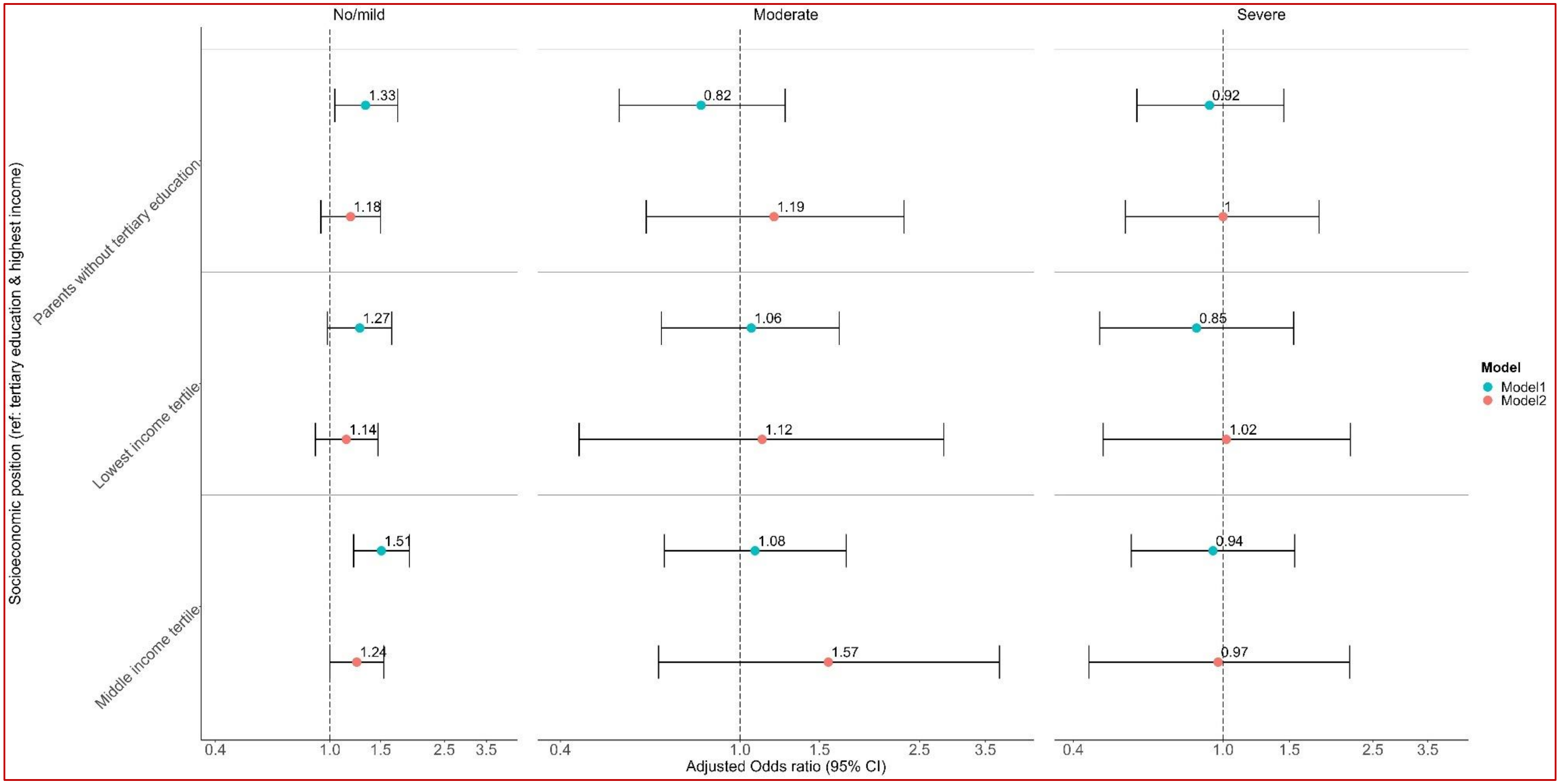

Fig s5. Comparison of estimates produced from the main analysis using self-reported mental health status (model1) and that using parent reported mental health status (model2). *Odds ratios (log-scale) for the association between SEP and MHC use. All models were adjusted for calendar year, region of residence, parent's country of birth, parental mental illness, and sex. Vertical bars represent 95% CIs.*

Table s9. Association between SEP and utilisation of any MHC (from 7th to 9th grade), moderated by adolescents' parent-reported mental health status.
Adjusted odds ratios with 95% confidence intervals given.

			No/mild symptoms	Moderate	Severe
**Type of service**	**SEP**	**SEP level**	**OR (95% CI)**	**OR (95% CI)**	**OR (95% CI)**
**Total (girls and boys)**					
Any MHC use	Tertiary education	No	1.18 (0.93, 1.50)	1.19 (0.62, 2.31)	1.00 (0.55, 1.80)
		Yes (ref)	1	1	1
	Household income	Low	1.14 (0.89, 1.47)	1.12 (0.44, 2.83)	1.02 (0.48, 2.18)
		Middle	1.24 (1.00, 1.54)*	1.57 (0.66, 3.76)	0.97 (0.44, 2.17)
		Highest (ref)	1	1	1
MHC use for Neuropsychiatric conditions	Tertiary education	No	**1.86 (1.32, 2.61)**	1.46 (0.55, 3.84)	1.52 (0.58, 3.97)
		Yes (ref)	1	1	1
	Household income	Low	**1.52 (1.10, 2.11)**	1.04 (0.25, 4.30)	1.38 (0.43, 4.41)
		Middle	**1.54 (1.17, 2.03)**	1.49 (0.40, 5.56)	1.03 (0.29, 3.65)
		Highest (ref)	1	1	1
MHC use for other mental disorders	Tertiary education	No	0.88 (0.65, 1.19)	0.85 (0.33, 2.18)	0.49 (0.21, 1.14)
		Yes (ref)	1	1	1
	Household income	Low	0.99 (0.72, 1.36)	0.92 (0.31, 2.71)	0.53 (0.18, 1.50)
		Middle	1.08 (0.81, 1.44)	0.96 (0.29, 3.13)	0.56 (0.20, 1.57)
		Highest (ref)	1	1	1
**Girls**					
Any MHC use	Tertiary education	No	0.99 (0.72, 1.37)	1.17 (0.49, 2.80)	1.23 (0.58, 2.64)
		Yes (ref)	1	1	1
	Household income	Low	0.88 (0.63, 1.23)	0.92 (0.31, 2.78)	1.18 (0.38, 3.62)
		Middle	0.98 (0.74, 1.31)	1.02 (0.34, 3.02)	1.27 (0.39, 4.19)
		Highest (ref)	1	1	1
MHC use for Neuropsychiatric conditions	Tertiary education	No	**2.67 (1.46, 4.90)**	1.61 (0.41, 6.34)	2.49 (0.70, 8.83)
		Yes (ref)	1	1	1

			No/mild symptoms	Moderate	Severe
**Type of service**	**SEP**	**SEP level**	**OR (95% CI)**	**OR (95% CI)**	**OR (95% CI)**
	Household income	Low	1.18 (0.63, 2.21)	0.43 (0.09, 2.11)	2.08 (0.30, 14.47)
		Middle	1.03 (0.59, 1.81)	0.60 (0.13, 2.73)	1.91 (0.25, 14.82)
		Highest (ref)	1	1	1
MHC use for other mental disorders	Tertiary education	No	0.73 (0.51, 1.05)*	1.03 (0.32, 3.29)	0.49 (0.18, 1.32)
		Yes (ref)	1	1	1
	Household income	Low	0.84 (0.58, 1.22)	1.55 (0.39, 6.17)	0.73 (0.19, 2.81)
		Middle	0.96 (0.70, 1.33)	1.07 (0.21, 5.53)	0.58 (0.13, 2.63)
		Highest (ref)	1	1	1
**Boys**					
Any MHC use	Tertiary education	No	**1.50 (1.05, 2.14)**	1.12 (0.40, 3.14)	0.83 (0.31, 2.22)
		Yes (ref)	1	1	1
	Household income	Low	**1.75 (1.23, 2.49)**	1.43 (0.24, 8.58)	0.86 (0.32, 2.27)
		Middle	**1.75 (1.26, 2.44)**	2.29 (0.42, 12.35)	0.79 (0.28, 2.22)
		Highest (ref)	1	1	1
MHC use for Neuropsychiatric conditions	Tertiary education	No	**1.57 (1.05, 2.34)**	2.13 (0.59, 7.74)	0.87 (0.19, 3.99)
		Yes (ref)	1	1	1
	Household income	Low	**1.63 (1.16, 2.29)**	3.83 (0.03, 496.72)	1.02 (0.22, 4.68)
		Middle	**1.67 (1.27, 2.19)**	5.10 (0.04, 619.59)	0.55 (0.07, 4.07)
		Highest (ref)	1	1	1
MHC use for other mental disorders	Tertiary education	No	1.43 (0.83, 2.48)	0.62 (0.11, 3.58)	0.59 (0.09, 3.66)
		Yes (ref)	1	1	1
	Household income	Low	1.77 (0.92, 3.41)	0.28 (0.04, 1.88)	0.25 (0.04, 1.38)
		Middle	1.71 (0.91, 3.22)	0.58 (0.12, 2.89)	0.34 (0.09, 1.36)
		Highest (ref)	1	1	1

- Reference categories are yes for parents with tertiary education & the highest household income tertile
- Different models were run for each SEP indicator
- Each model was adjusted for calendar year, region of residence, parent's country of birth, & parental mental illness (and adjusted for sex for the Total model)

			No/mild symptoms	Moderate	Severe
**Type of service**	**SEP**	**SEP level**	**OR (95% CI)**	**OR (95% CI)**	**OR (95% CI)**

- Statistically significant estimates highlighted in bold font
- * Borderline significance
- SEP = Socioeconomic position, MHC = Mental health care; OR=Odds ratio

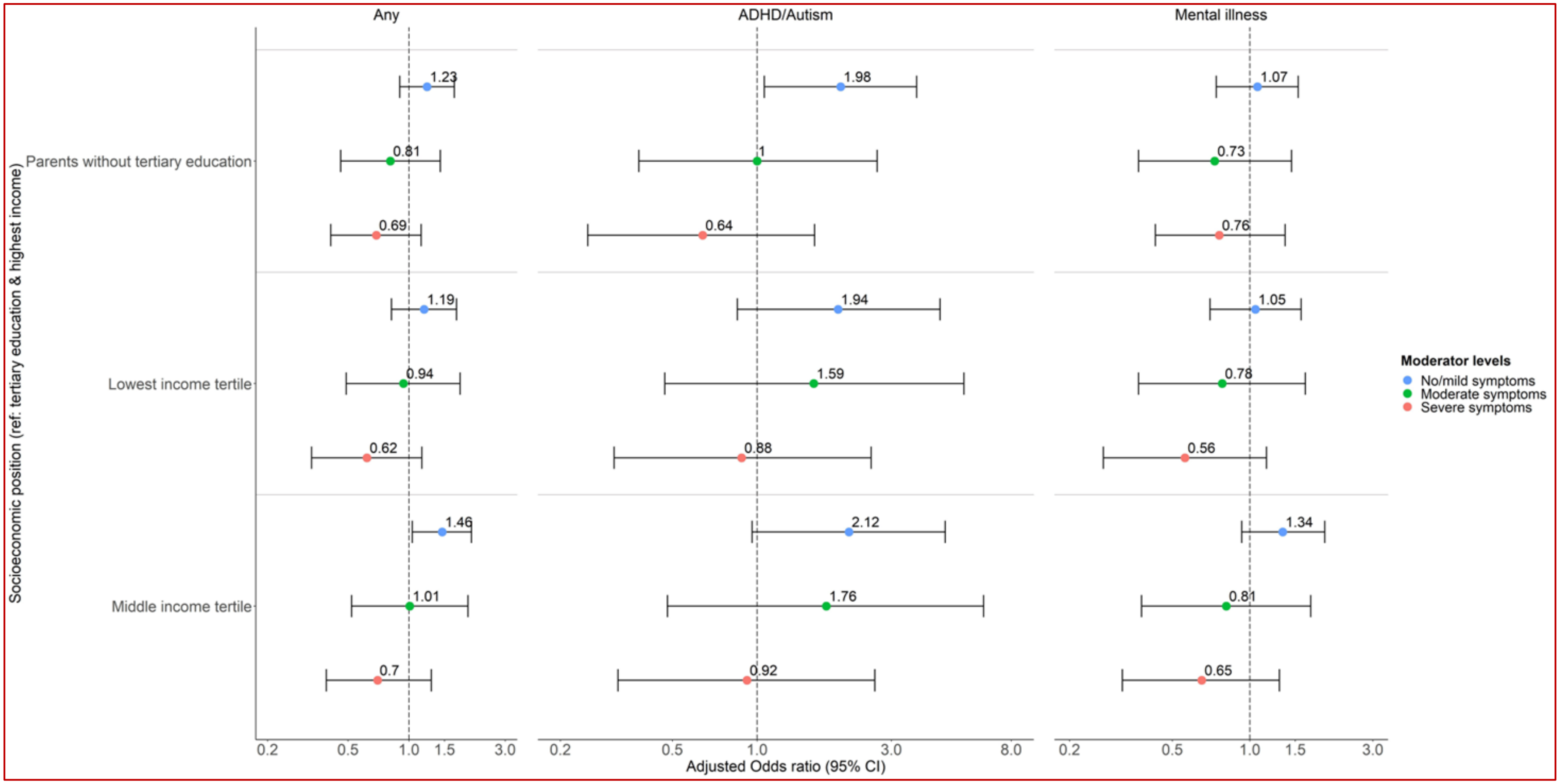

Fig s6. Adjusted odds ratios (log-scale) of the association between SEP and utilisation of any MHC at least once, excluding adolescents who utilised MHC in the previous 6 months before follow-up (i.e., incident utilisation). *Association moderated by adolescent's self-reported mental health status. All models were adjusted for calendar year, region of residence, parent's country of birth, parental mental illness, and sex. Vertical bars represent 95% CIs.*

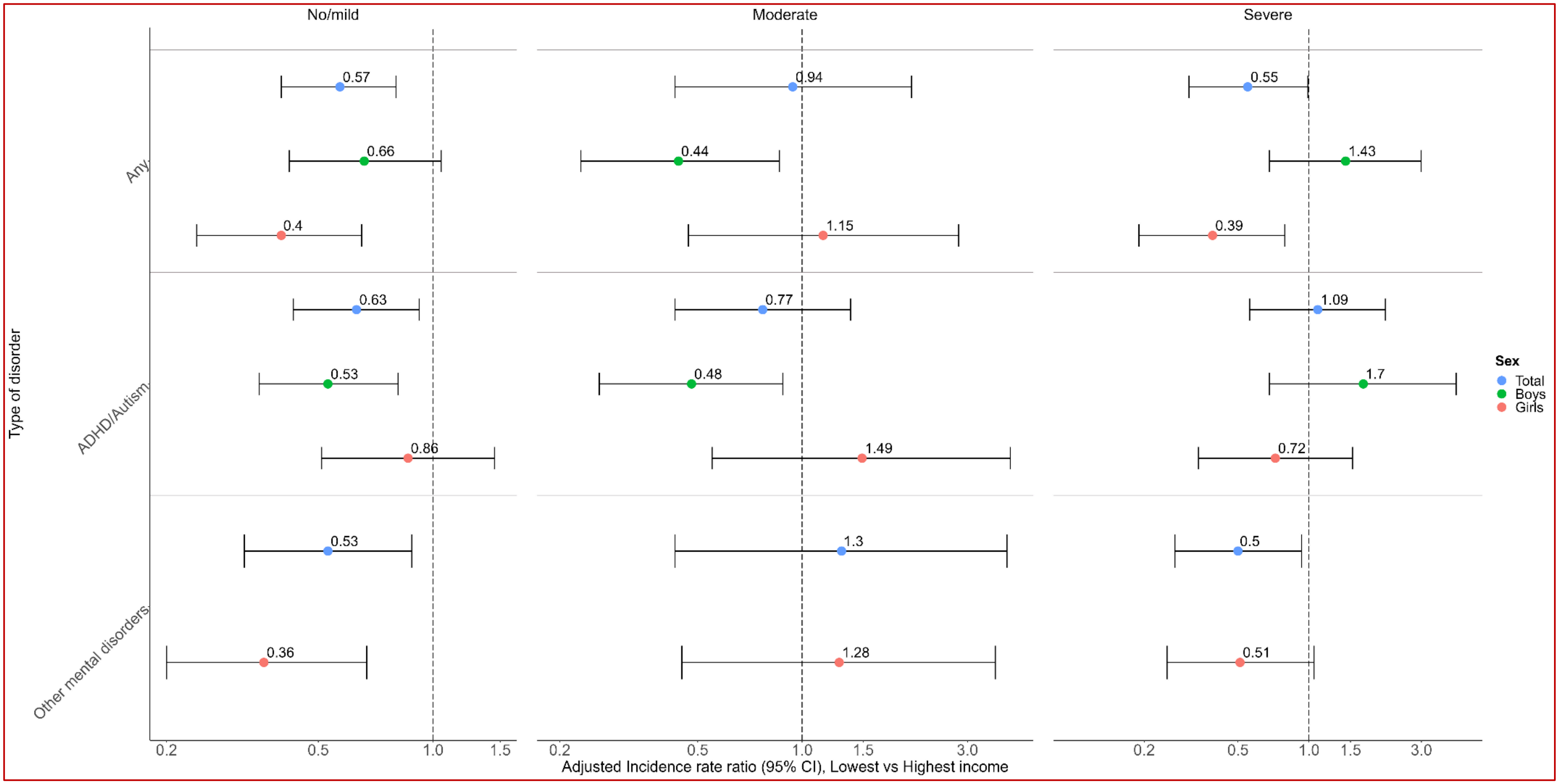

Fig s7. Adjusted incidence rate ratios (log-scale) for the moderated association between household income and number of outpatient visits for 12 months following each survey. *Association moderated by adolescents' self-reported mental health status at each grade. All models were adjusted for calendar year, region of residence, parent's country of birth, parental mental illness (and adjusted for sex in the unstratified model, Total). Vertical bars represent 95% CIs. Note: lowest tertile vs Highest tertile, see table s10 for comparison between middle tertile and highest tertile.*

Table s10. Association between SEP and number of outpatient visits to secondary care (from 7th to 9th grade), moderated by adolescents' self-reported mental health status. *Adjusted Incidence rate ratios with 95% confidence intervals given.*

			All[a]	No/mild symptoms	Moderate	Severe
**Type of service**	**SEP**	**SEP level**	**IRR (95% CI)**	**IRR (95% CI)**	**IRR (95% CI)**	**IRR (95% CI)**
**Total (girls and boys)**						
Outpatient care (N=992)	Tertiary education	No	0.81 (0.63, 1.05)*	**0.71 (0.52, 0.96)**	1.04 (0.55, 1.96)	0.88 (0.54, 1.46)
		Yes (ref)	1	1	1	1
	Household income	Low	**0.64 (0.47, 0.89)**	**0.57 (0.40, 0.80)**	0.94 (0.43, 2.07)	**0.55 (0.31, 0.99)**
		Middle	0.88 (0.65, 1.18)	0.84 (0.58, 1.23)	0.83 (0.49, 1.41)	0.94 (0.52, 1.69)
		Highest (ref)	1	1	1	1
MHC use for Neuropsychiatric conditions (N=512)	Tertiary education	No	0.99 (0.77, 1.29)	0.92 (0.68, 1.23)	1.40 (0.91, 2.16)	0.93 (0.58, 1.47)
		Yes (ref)	1	1	1	1
	Household income	Low	0.75 (0.55, 1.03)*	**0.63 (0.43, 0.92)**	0.77 (0.43, 1.38)	1.09 (0.56, 2.11)
		Middle	0.78 (0.59, 1.04)*	0.76 (0.53, 1.09)	0.66 (0.39, 1.12)	0.92 (0.49, 1.71)
		Highest (ref)	1	1	1	1
MHC use for other mental disorders (N=480)	Tertiary education	No	0.79 (0.53, 1.19)	0.68 (0.40, 1.16)	0.68 (0.32, 1.46)	1.18 (0.54, 2.57)
		Yes (ref)	1	1	1	1
	Household income	Low	**0.65 (0.42, 0.99)**	**0.53 (0.32, 0.88)**	1.30 (0.43, 3.90)	**0.50 (0.27, 0.93)**
		Middle	0.96 (0.63, 1.46)	0.92 (0.48, 1.74)	1.39 (0.80, 2.41)	0.80 (0.36, 1.78)
		Highest (ref)	1	1	1	1
**Girls**						
Outpatient care (N=449)	Tertiary education	No	0.81 (0.56, 1.18)	0.61 (0.36, 1.01)*	1.13 (0.53, 2.43)	0.93 (0.50, 1.74)
		Yes (ref)	1	1	1	1
	Household income	Low	**0.56 (0.36, 0.87)**	**0.40 (0.24, 0.65)**	1.15 (0.47, 2.83)	**0.39 (0.19, 0.79)**
		Middle	0.89 (0.61, 1.30)	0.75 (0.46, 1.24)	1.23 (0.69, 2.20)	0.82 (0.43, 1.57)
		Highest (ref)	1	1	1	1
MHC use for Neuropsychiatric conditions (N=172)	Tertiary education	No	1.05 (0.70, 1.55)	0.93 (0.63, 1.39)	1.75 (0.93, 3.30)	0.87 (0.49, 1.56)

			All[a]	No/mild symptoms	Moderate	Severe
**Type of service**	**SEP**	**SEP level**	**IRR (95% CI)**	**IRR (95% CI)**	**IRR (95% CI)**	**IRR (95% CI)**
		Yes (ref)	1	1	1	1
	Household income	Low	0.98 (0.60, 1.60)	0.86 (0.51, 1.45)	1.49 (0.55, 3.99)	0.72 (0.34, 1.53)
		Middle	1.01 (0.64, 1.60)	1.03 (0.58, 1.83)	1.09 (0.42, 2.79)	0.89 (0.41, 1.91)
		Highest (ref)	1	1	1	1
MHC use for other mental disorders (N=371)	Tertiary education	No	0.84 (0.51, 1.38)	0.70 (0.35, 1.39)	0.70 (0.31, 1.59)	1.29 (0.53, 3.14)
		Yes (ref)	1	1	1	1
	Household income	Low	**0.57 (0.36, 0.93)**	**0.36 (0.20, 0.67)**	1.28 (0.45, 3.61)	0.51 (0.25, 1.05)*
		Middle	0.93 (0.61, 1.42)	0.73 (0.40, 1.32)	1.49 (0.88, 2.55)	0.84 (0.36, 1.99)
		Highest (ref)	1	1	1	1
**Boys**						
Outpatient care (N=449)	Tertiary education	No	0.79 (0.58, 1.07)	0.77 (0.54, 1.09)	0.85 (0.48, 1.49)	0.81 (0.44, 1.52)
		Yes (ref)	1	1	1	1
	Household income	Low	0.73 (0.49, 1.08)	0.66 (0.42, 1.05)*	**0.44 (0.23, 0.86)**	1.43 (0.68, 2.99)
		Middle	0.86 (0.54, 1.35)	0.88 (0.50, 1.55)	**0.44 (0.23, 0.84)**	1.03 (0.51, 2.07)
		Highest (ref)	1	1	1	1
MHC use for Neuropsychiatric conditions (N=340)	Tertiary education	No	0.92 (0.66, 1.27)	0.92 (0.63, 1.33)	0.95 (0.55, 1.64)	0.89 (0.46, 1.71)
		Yes (ref)	1	1	1	1
	Household income	Low	**0.65 (0.44, 0.94)**	**0.53 (0.35, 0.81)**	**0.48 (0.26, 0.88)**	1.70 (0.68, 4.21)
		Middle	0.72 (0.51, 1.02)*	0.68 (0.46, 1.01)*	0.56 (0.31, 1.00)*	1.05 (0.46, 2.44)
		Highest (ref)	1	1	1	1
MHC use for other mental disorders (N=109)	Tertiary education	No	-	-	-	-
		Yes (ref)	1	1	1	1
	Household income	Low	-	-	-	-
		Middle	-	-	-	-
		Highest (ref)	1	1	1	1

- Reference categories are yes for parents with tertiary education & the highest household income tertile

			All[a]	No/mild symptoms	Moderate	Severe
**Type of service**	**SEP**	**SEP level**	**IRR (95% CI)**	**IRR (95% CI)**	**IRR (95% CI)**	**IRR (95% CI)**

- Different models were run for each SEP indicator
- Each model was adjusted for calendar year, region of residence, parent's country of birth, & parental mental illness (and adjusted for sex for the Total model)
- (a) Estimates under all, represent the association between SEP and number of outpatient visits for all adolescents regardless of their mental health status (adjusted for mental health status + other covariates)
- Statistically significant estimates highlighted in bold font
- * borderline significance
- (–) models did not converge.
- SEP = Socioeconomic position, MHC = Mental health care; IRR=Incidence rate ratio

Table s11. Association between SEP and number of outpatient visits to secondary care (from 7th to 9th grade), moderated by adolescents' parent-reported mental health status. *Adjusted Incidence rate ratios with 95% confidence intervals given.*

			All[a]	No/mild symptoms	Moderate	Severe
**Type of service**	**SEP**	**SEP level**	**IRR (95% CI)**	**IRR (95% CI)**	**IRR (95% CI)**	**IRR (95% CI)**
**Total (girls and boys)**						
Outpatient care (N=992)	Tertiary education	No	0.82 (0.63, 1.07)	0.88 (0.65, 1.21)	0.90 (0.51, 1.56)	0.63 (0.39, 1.01)*
		Yes (ref)	1	1	1	1
	Household income	Low	**0.74 (0.55, 0.99)**	**0.67 (0.49, 0.92)**	0.51 (0.26, 1.02)*	1.11 (0.61, 2.02)
		Middle	0.91 (0.68, 1.23)	0.96 (0.67, 1.38)	1.00 (0.52, 1.91)	0.71 (0.41, 1.25)
		Highest (ref)	1	1	1	1
MHC use for Neuropsychiatric conditions (N=512)	Tertiary education	No	0.94 (0.73, 1.20)	1.00 (0.75, 1.34)	1.14 (0.71, 1.84)	0.79 (0.55, 1.14)
		Yes (ref)	1	1	1	1
	Household income	Low	0.80 (0.59, 1.08)	0.79 (0.55, 1.12)	0.67 (0.37, 1.20)	0.85 (0.49, 1.45)
		Middle	0.83 (0.62, 1.10)	0.80 (0.58, 1.11)	1.11 (0.67, 1.83)	0.71 (0.40, 1.24)
		Highest (ref)	1	1	1	1
MHC use for other mental disorders (N=480)	Tertiary education	No	0.79 (0.53, 1.17)	0.84 (0.54, 1.31)	0.46 (0.21, 1.00)*	0.62 (0.23, 1.65)
		Yes (ref)	1	1	1	1
	Household income	Low	0.80 (0.54, 1.19)	0.72 (0.48, 1.07)	0.54 (0.26, 1.15)	2.38 (0.85, 6.68)
		Middle	0.98 (0.64, 1.50)	1.03 (0.64, 1.66)	0.63 (0.21, 1.90)	0.92 (0.51, 1.68)

			All[a]	No/mild symptoms	Moderate	Severe
**Type of service**	**SEP**	**SEP level**	**IRR (95% CI)**	**IRR (95% CI)**	**IRR (95% CI)**	**IRR (95% CI)**
		Highest (ref)	1	1	1	1
**Girls**						
Outpatient care (N=449)	Tertiary education	No	0.84 (0.56, 1.26)	0.92 (0.59, 1.44)	0.96 (0.40, 2.30)	0.53 (0.25, 1.15)
		Yes (ref)	1	1	1	1
	Household income	Low	**0.64 (0.42, 0.97)**	**0.55 (0.35, 0.86)**	**0.26 (0.12, 0.54)**	1.76 (0.70, 4.43)
		Middle	0.91 (0.62, 1.34)	1.02 (0.66, 1.56)	0.62 (0.28, 1.41)	0.90 (0.41, 1.97)
		Highest (ref)	1	1	1	1
MHC use for Neuropsychiatric conditions (N=172)	Tertiary education	No	1.00 (0.67, 1.50)	1.08 (0.62, 1.88)	1.17 (0.50, 2.71)	0.86 (0.51, 1.47)
		Yes (ref)	1	1	1	1
	Household income	Low	0.83 (0.51, 1.34)	0.82 (0.41, 1.63)	0.67 (0.24, 1.89)	0.96 (0.42, 2.20)
		Middle	1.07 (0.68, 1.69)	1.09 (0.58, 2.04)	1.08 (0.46, 2.53)	1.09 (0.48, 2.50)
		Highest (ref)	1	1	1	1
MHC use for other mental disorders (N=371)	Tertiary education	No	0.81 (0.48, 1.36)	0.87 (0.49, 1.51)	0.62 (0.27, 1.42)	0.55 (0.19, 1.62)
		Yes (ref)	1	1	1	1
	Household income	Low	0.69 (0.44, 1.06)	0.64 (0.41, 1.00)*	**0.25 (0.13, 0.49)**	2.40 (0.77, 7.45)
		Middle	0.90 (0.58, 1.40)	0.97 (0.60, 1.56)	0.54 (0.20, 1.47)	0.83 (0.36, 1.92)
		Highest (ref)	1	1	1	1
**Boys**						
Outpatient care (N=449)	Tertiary education	No	0.79 (0.61, 1.04)*	0.79 (0.58, 1.07)	0.84 (0.42, 1.68)	0.78 (0.50, 1.22)
		Yes (ref)	1	1	1	1
	Household income	Low	0.89 (0.62, 1.28)	0.94 (0.59, 1.49)	1.10 (0.48, 2.56)	0.79 (0.46, 1.37)
		Middle	0.91 (0.58, 1.45)	0.98 (0.51, 1.91)	1.58 (0.73, 3.38)	0.58 (0.32, 1.05)*
		Highest (ref)	1	1	1	1
Care for Neuropsychiatric conditions (N=340)	Tertiary education	No	0.91 (0.67, 1.22)	0.95 (0.69, 1.30)	1.16 (0.59, 2.25)	0.76 (0.48, 1.21)
		Yes (ref)	1	1	1	1
	Household income	Low	0.74 (0.52, 1.06)	0.75 (0.52, 1.10)	0.63 (0.32, 1.23)	0.74 (0.41, 1.35)

Type of service	SEP	SEP level	All[a] IRR (95% CI)	No/mild symptoms IRR (95% CI)	Moderate IRR (95% CI)	Severe IRR (95% CI)
		Middle	0.73 (0.52, 1.02)*	**0.69 (0.48, 0.99)**	1.17 (0.64, 2.13)	0.55 (0.29, 1.05)*
		Highest (ref)	1	1	1	1
MHC use for other mental disorders (N=109)	Tertiary education	No	0.68 (0.40, 1.16)	-	-	-
		Yes (ref)	1	1	1	1
	Household income	Low	-	-	-	-
		Middle	-	-	-	-
		Highest (ref)	1	1	1	1

- Reference categories are yes for parents with tertiary education & the highest household income tertile
- Different models were run for each SEP indicator; SEP = Socioeconomic position
- Each model was adjusted for calendar year, region of residence, parent's country of birth, & parental mental illness (and adjusted for sex for the Total model)
- (a) Estimates under All, represent the association between SEP and number of outpatient visits for all adolescents regardless of their mental health status (adjusted for mental health status + other covariates)
- Statistically significant estimates highlighted in bold font
- * Borderline significance
- (–) models did not converge
- SEP = Socioeconomic position, MHC = Mental health care; IRR=Incidence rate ratio
